# CENH3-GFP: a visual marker for gametophytic and somatic ploidy determination in *Arabidopsis thaliana*

**DOI:** 10.1186/s12870-015-0700-5

**Published:** 2016-01-05

**Authors:** Nico De Storme, Burcu Nur Keçeli, Linda Zamariola, Geert Angenon, Danny Geelen

**Affiliations:** In vitro Biology and Horticulture, Department of Plant Production, Faculty of Bioscience Engineering, Ghent University, Coupure Links 653, B-9000 Ghent, Belgium; Institute for Molecular Biology and Biotechnology, VUB, Pleinlaan 2, B-1050 Brussels, Belgium

**Keywords:** CENH3, Centromere, Arabidopsis, Ploidy analysis, Meiosis, Endomitosis

## Abstract

**Background:**

The in vivo determination of the cell-specific chromosome number provides a valuable tool in several aspects of plant research. However, current techniques to determine the endosystemic ploidy level do not allow non-destructive, cell-specific chromosome quantification. Particularly in the gametophytic cell lineages, which are physically encapsulated in the reproductive organ structures, direct in vivo ploidy determination has been proven very challenging. Using *Arabidopsis thaliana* as a model, we here assess the applicability of recombinant *CENH3-GFP* reporters for the labeling of the cell’s chromocenters and for the monitoring of the gametophytic and somatic chromosome number in vivo.

**Results:**

By modulating expression of a *CENH3-GFP* reporter cassette using different promoters, we isolated two reporter lines that allow for a clear and highly specific labeling of centromeric chromosome regions in somatic and gametophytic cells respectively. Using polyploid plant series and reproductive mutants, we demonstrate that the *pWOX2*-*CENH3-GFP* recombinant fusion protein allows for the determination of the gametophytic chromosome number in both male and female gametophytic cells, and additionally labels centromeric regions in early embryo development. Somatic centromere labeling through *p35S-CENH3-GFP* shows a maximum of ten centromeric dots in young dividing tissues, reflecting the diploid chromosome number (2x = 10), and reveals a progressive decrease in GFP foci frequency throughout plant development. Moreover, using chemical and genetic induction of endomitosis, we demonstrate that *CENH3*-mediated chromosome labeling provides an easy and valuable tool for the detection and characterization of endomitotic polyploidization events.

**Conclusions:**

This study demonstrates that the introgression of the *pWOX2*-*CENH3-GFP* reporter construct in *Arabidopsis thaliana* provides an easy and reliable methodology for determining the chromosome number in developing male and female gametes, and during early embryo development. Somatically expressed CENH3-GFP reporters, on the other hand, constitute a valuable tool to quickly determine the basic somatic ploidy level in young seedlings at the individual cell level and to detect and to quantify endomitotic polyploidization events in a non-destructive, microscopy-based manner.

**Electronic supplementary material:**

The online version of this article (doi:10.1186/s12870-015-0700-5) contains supplementary material, which is available to authorized users.

## Background

The exact quantification of chromosome number and ploidy level is an important aspect of genetic, molecular and evolutionary research in plants. Particularly in research topics covering genomic stability and integrity, somaclonal variation, chromosome segregation, aneuploidy and polyploidy, the accurate assessment of the plant’s chromosome number, either within an organ or a specific cell type, is essential for phenotypic characterization [1–4]. Quantification of the somatic chromosome number not only provides information about the basic ploidy level, but also allows for the detection of endosystemic polyploidy and putative aneuploidy [5, 6]. Similarly, in the reproductive cell lineage, determination of the chromosome number allows for gametophytic ploidy analysis and the associated assessment of meiotic cell division integrity [7]. Particularly concerning polyploid and aneuploid gamete formation [8, 9], the quantification of chromosome number in developing mega- and microspores is highly relevant and essential for correct phenotypic assessment.

During the last decades, several techniques have been developed that allow for the determination of organ- or plant-specific ploidy levels; including DNA flow cytometry [10–12], chromosome spreading and fluorescent *in situ* hybridization (FISH) [13–15]. However, none of these methodologies enables in vivo chromosome quantification on a single-cell level.

DNA flow cytometry is a well-known technique that is commonly used to analyze the ploidy level of large cell populations [16], however, the destructive sample preparation (e.g. nuclear cell suspension) impairs any in vivo cell- or tissue-specific ploidy determination [17]. Moreover, DNA flow cytometry involves high throughput screening and inherently confers a basic level of background interference, making it not suitable for the detection of infrequent ploidy aberrations or minor alterations in chromosome number [12]. For the same reason, DNA flow cytometry cannot be used for the ploidy analysis of cell types which are embedded in surrounding tissue (e.g., vascular cell layers, egg cells, early embryonic cells).

Besides DNA flow cytometry, the cell’s chromosome number can also be determined by cytological approaches, including chromosome spreading and FISH. In the chromosome spreading methodology, biological material is fixed, hydrolyzed, spread on a slide and stained using DNA-specific dyes, allowing for a visual chromosome or DNA quantification on a single cell basis [18]. However, since accurate chromosome quantification requires a fully condensed chromosome state, only actively dividing cells can be assessed, largely impairing the biological application range [14, 15, 19]. In the FISH approach, this issue is overcome by the use of chromosome-specific probes [20–22]. These oligos typically recognize and label specific chromosomal regions or sequences, allowing accurate determination of chromosome number in both condensed and non-condensed cells [23–25]. However, since this technique requires a specific probe (or probe combination) for each chromosome [26], ploidy determination through FISH is laborious and expensive and is therefore only used in species with low chromosome numbers. In the search for a single probe that indiscriminately labels all chromosomes, oligos that specifically recognize the centromeric DNA repeat have been found to be highly promising for the accurate determination of chromosome number [27, 28]. Moreover, with the recent identification of centromere-specific proteins in plants (e.g., histones, kinetochore proteins) [29–32], immunocytology can also be used to detect and to quantify centromeric regions in individual plant cells [33–35]. However, although FISH and immunocytology both allow accurate chromosome quantification, the associated sample preparation protocol generally requires fixation and digestion, largely disrupting the spatial intra-organ cellular arrangement and cell identity. Hence, these techniques interfere with a proper cell type characterization and thus do not allow for a proper in vivo determination of the absolute chromosome number.

In all eukaryotes, centromeres are essential for the proper loading and nucleation of kinetochore protein complexes and the associated attachment of spindle microtubules during chromosome division. Despite their universal function, centromeric DNA sequences show a high variability in size and structure among all eukaryotic taxa, ranging from short unique centromere DNA sequences in budding yeast (e.g., 125 bp in *S. cerevisiae*) to whole-chromosome spanning genome regions in *C. elegans* [36]. In plants, similar to animals, centromeric chromosome regions are constituted by large DNA tracts (several megabases) consisting of multiple copies of simple tandem repeat arrays, often harboring a particular type of retrotransposon [37–40].

In contrast to the extreme diversity observed at the DNA level, centromeric regions in all eukaryotic taxa contain one specific histone H3 variant; e.g. CENH3 [41]. This histone type is referred to as CSE in fungi, CENP-A in metazoans, CID in Drosophila and HTR12 or CENH3 in plants. In all species studied, CENH3 typically replaces the conventional nucleosomal histone H3 at the centromeric core [29], where it forms a structurally distinct chromatin domain that defines the centromeric chromosome region [42, 43]. In addition, CENH3 recruits the kinetochore multiprotein complex to the centromere, ensuring correct meiotic and mitotic chromosome segregation [41, 44, 45]. As such, the presence of histone CENH3 is generally considered the most credible factor defining centromere identity.

In the search for a gamete-specific in vivo ploidy marker, we here describe the characterization of recombinant *CENH3-GFP* constructs in *Arabidopsis thaliana* development, and particularly in male and female reproduction. We demonstrate that *CENH3-GFP* confers distinct labeling of centromeres in several tissue types, including developing gametes, and show that this allows for a tissue- or cell-specific chromosome quantification. We particularly focus on *pWOX2-CENH3-GFP* as this construct is specifically expressed in male and female gametophytic cells. Based on a ploidy series, we found that *pWOX2-CENH3-GFP*-mediated ploidy analysis at the early mega- and microspore stage closely corresponds to meiotic chromosome segregation data and hence provides a quick and reliable method to assess gametophytic ploidy and meiotic stability. We additionally show that somatically expressed *CENH3-GFP* also labels centromeres and thus allows for a rapid cell-specific chromosome quantification in somatic tissue types; including embryo’s, root tips, leaf cells and developing flower organs. As such, *CENH3*-mediated centromere labeling constitutes a unique methodology to detect and to quantify endomitotic endoploidy in vivo.

## Results

### Modulating expression of *CENH3-GFP* in male and female gametophyte development

To validate the use of the centromere-specific protein CENH3 in quantifying the cell-specific number of centromeres in male and female sporo- and gametogenesis, a set of *CENH3-GFP* reporter constructs was generated. Specific developmental expression and variability in signal intensity was obtained by fusing a *CENH3-GFP* cassette to several meiosis- and gamete-specific promoters (Table 1). ASY2 and MSH4 are involved in synapsis and recombination, respectively, and are therefore suggested to be expressed in meiotic prophase I. JASON (JAS) and AtPS1 are required for the perpendicular orientation of metaphase II spindles in male meiosis, suggesting an MII-specific expression pattern. The *LAT52* and *WOX2* promoters were included to specifically express CENH3-GFP in mature pollen and in the female germ lineage, respectively. Finally, as a control, the 35S promotor was also used to monitor CENH3-GFP patterning under constitutive expression. Following cloning and floral dip transformation, T1 lines were grown on selective medium (e.g. kanamycin) and resistant lines were microscopically assessed for GFP expression in several stages of plant development, including somatic and reproductive organs.

**Table 1:** Overview of CENH3-GFP constructs and their developmental expression profile

CENH3-GFP reporter construct		GFP expression profile									
Promotor	Expected expression profile	# T1 lines	Root	Leaf		Petal	MC	MS	Pollen	Ovule	ES
pro35S	constitutive	40	+	+	+		_−_	_−_	_−_	+	_−_
proASY2	prophase I	26	n.a.	n.a.	-		-	+	-	-	-
proMSH4	prophase I	37	-	n.a.	+		-	+	-	+	-
proJAS	male metaphase II	34	-	n.a.	-		+	-	-	-	-
proAtPS1	male metaphase II	15	+	-	+		+	+	-	+	-
proWOX2	egg cell and embryo	42	+	-	-		-	+	+	-	+
proLAT52	mature pollen	14	n.a.	n.a.	n.a.		-	-	+	-	-

n.a.: not analysed

**Fig. 1:**
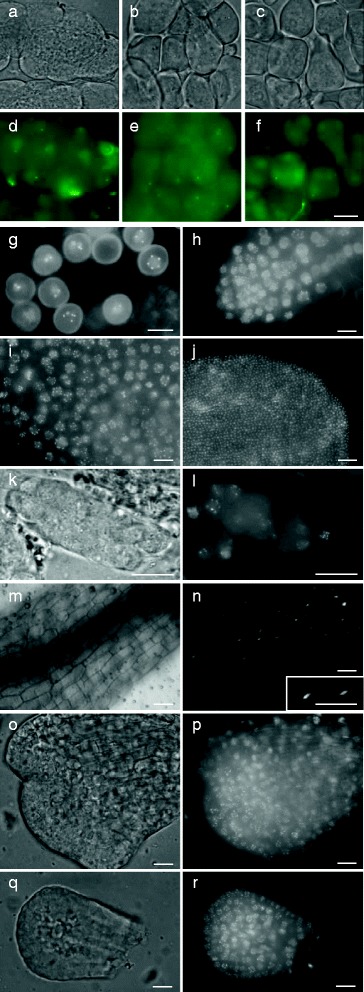
Modulating *CENH3-GFP* expression in Arabidopsis using different promoters. **a**-**f** Bright field and GFP fluorescent imaging in wild type PMCs showing autofluorescent dots at meiotic initiation **a** and **d**, prophase I **b** and **e** and tetrad stage **c** and **f**. **g**-**r** Promoter-dependent expression and localization of *CENH3-GFP* in somatic and reproductive organs in Arabidopsis. **g** *pASY2-CENH3-GFP* fluorescent dots in uninuclear microspores. **h**-**j** Expression of *pAtPS1-* and *p35S-CENH3-GFP* in respectively roots **h** and leaves **i** of ten-day-old seedlings. Scale bars, 10 μm. **j** *pAtPS1-CENH3-GFP* expression in mature petals. Scale bar, 50 μm. **k**-**l** GFP fluorescent imaging of *pAtPS1-CENH3-GFP* premeiotic cells in male sporogenesis. **m**-**r** GFP fluorescence in *p35S-CENH3-GFP* hypocotyls **m**-**n**, flower meristems **o**-**p** and young petals **q**-**r**. Scale bars, 10 μm, except for hypocotyls; 25 μm

By monitoring background fluorescence, we found that pollen mother cells (PMCs) in non-recombinant Arabidopsis wild type plants display autofluorescent dots in all stages of meiocyte development. More specifically, control meiocytes consistently showed one fluorescent dot at the start of meiosis, which persisted throughout meiosis, and resulted in tetrads containing four haploid spores, that each display one green fluorescent dot (Fig. 1a-f). The fluorescent foci were also present in newly formed microspores and subsequently disappeared at the mid-uninuclear stage. In contrast, pre-meiotic cells and cells from other flower tissues did not show autofluorescence under the excitation settings used. We currently do not know whether the observed autofluorescent signal in developing meiocytes is associated with any organelle or other cellular structure but clearly it interferes with GFP-labeled protein analysis and, as such, prevents the in vivo analysis of male meiotic chromosome behavior and ploidy determination using GFP-labeled (centromeric) proteins. However, since GFP autofluorescence is not observed in the stages following meiosis, CENH3-GFP-mediated centromere labeling can be used to quantify chromosomes in the gametophytic cell lineage.

The expression profile and centromere-specific labeling of the different CENH3-GFP reporter constructs made was analyzed at different developmental stages. Microscopic analysis revealed that, in contrast to the meiosis-specific function of ASY2 and MSH4, the corresponding CENH3-GFP constructs are not expressed in developing PMCs, but instead show a variable expression covering both somatic and reproductive tissues (Table 1). More specifically, *pASY2*-*CENH3-GFP* only shows expression in uninuclear microspores (Fig. 1g), whereas *pMSH4*-mediated expression is observed in several types of plant tissues; including roots, leaves and petals (Fig. 1h-j). When using *pJAS* or *pAtPS1*, *CENH3-GFP* is expressed in pre-meiotic PMCs, but not during the meiotic cell division (Fig. 1l). Moreover, in contrast to *pJAS-CENH3-GFP*, which is only expressed in pre-meiotic cells, *pAtPS1*-*CENH3-GFP* also appears in other somatic tissues; such as roots, petals and microspores (Table 1). Expression of CENH3-GFP under control of the 35S promotor showed a constitutive expression pattern, with GFP signals occurring in all somatic plant tissues, including roots, leaves and petals. In contrast, in reproductive tissues, p35S-CENH3-GFP is not expressed, impairing its use as a gametophytic centromere marker.

In all but one line, CENH3-GFP exhibits a nuclear localization pattern with a small number (e.g. 4–10) of bright fluorescent dots scattered in the nuclear region (Fig. 1g-l). As the only exception, CENH3-GFP expression driven by the pollen-specific LAT52 promoter displays strong fluorescence in both the cytoplasm and the vegetative nucleus of mature pollen (Additional file 1: Figure S1). We therefore conclude that *pLAT52*-*CENH3-GFP* expression in mature pollen is too abundant and hence confounds assessment of centromeric GFP signals. In contrast, pWOX2-CENH3-GFP results in a clearly dotted nuclear-localized fluorescent signal that is exclusively expressed in developing male and female gametophytic cells (e.g. microspores, pollen and embryo sacs), suggesting for the specific labeling of centromeric regions in both types of reproductive cell lineages (Table 1).

Except for the cytoplasmic fluorescence in *pLAT52::CENH3-GFP* pollen, all CENH3-GFP reporter lines exhibited a nuclear-specific GFP fluorescence signal, typically showing a specific localization pattern depending on the type of tissue. Fully differentiated cells in organs like the hypocotyl, mature root parts, etc. show a more or less homogenous fluorescence signal at the nuclear region (Fig. 1n). In contrast, mitotically active cells in root tips, flower meristems and emerging petals accumulate CENH3-GFP in a small number of fluorescent dots in the nuclear region (Fig. 1o-r). As this pattern is also observed in other centromeric labeling approaches (e.g. FISH with centromere-specific 180 bp repeats) [27] and CENH3 has repeatedly been found to localize to the cell’s chromocenters [30, 33, 34], we conclude that the CENH3-GFP dots correspond to centromeric regions. This is supported by the observation that the CENH3-GFP foci in all lines localize to the intensively DAPI-stained nuclear chromocenters (Additional file 2: Figure S2), indicating that CENH3-GFP can be used as an in vivo marker to label the cell’s centromeric regions.

To validate the centromere-specific accumulation of CENH3-GFP, a set of complementation tests was performed by introgressing both *pWOX2*- and *p35S-CENH3-GFP* in the *cenh3-1*^*−/−*^ background. As the homozygous null allele *cenh3-1*^*−/−*^ is embryo lethal [46], complementation consisted of recovering viable *cenh3-1*^*−/−*^ plants from a heterozygous *cenh3-1/CENH3* parent by the introgression of a specific CENH3-GFP construct (by intercrossing). Strikingly, for all constructs tested, not a single *cenh3-1*^*−/−*^ plant was retrieved (Additional file 3: Table S1). Moreover, seed set analysis in *cenh3-1/CENH3* T1 plants hemizygous for the *CENH3-GFP* transgene revealed a seed abortion ratio closely matching 1 to 3, indicating that *cenh3-1*^*−/−*^ embryo lethality is not complemented (Additional file 4: Figure S3). The inability to complement is most likely caused by the specific configuration of the C-terminal tagged CENH3-GFP fusion protein, which in previous studies has already been found to allow centromeric loading of CENH3, but to impair its function in somatic cell division [33, 46, 47]. Thus, the CENH3-GFP fusion proteins in this study can be used for in vivo labeling of centromeric regions, as long as the endogenous CENH3 protein is present.

### *pWOX2::CENH3-GFP* labels centromeric regions in male and female gametogenesis

**Fig. 2:**
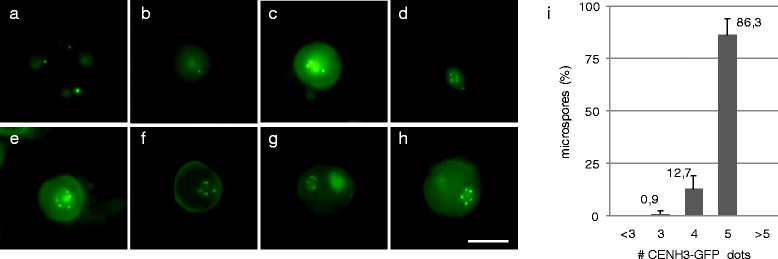
*CENH3-GFP* driven by *pWOX2* labels centromeres in male gametogenesis. **a**-**h** Expression and centromere-specific localization of the pWOX2-CENH3-GFP protein during male gametophyte development; meiotic tetrad stage **a**, early uninuclear microspore **b**, mid-uninuclear microspore **c**, late uninuclear microspore **d** and **e**, first mitotic division with doublet CENH3-GFP dots **f**, binuclear microspore stage **g** and **h**. Images are processed z-stack files. Bright fluorescent dots in **a** and **b** are also observed in wild type. Scale bar, 10 μm. **i** Frequency distribution of male spores (*n* = 383) depending on the number of CENH3-GFP fluorescent dots at the late uninuclear microspore stage. Error bars represent standard deviation (of three independent analyses of at least 100 spores)

**Fig. 3:**
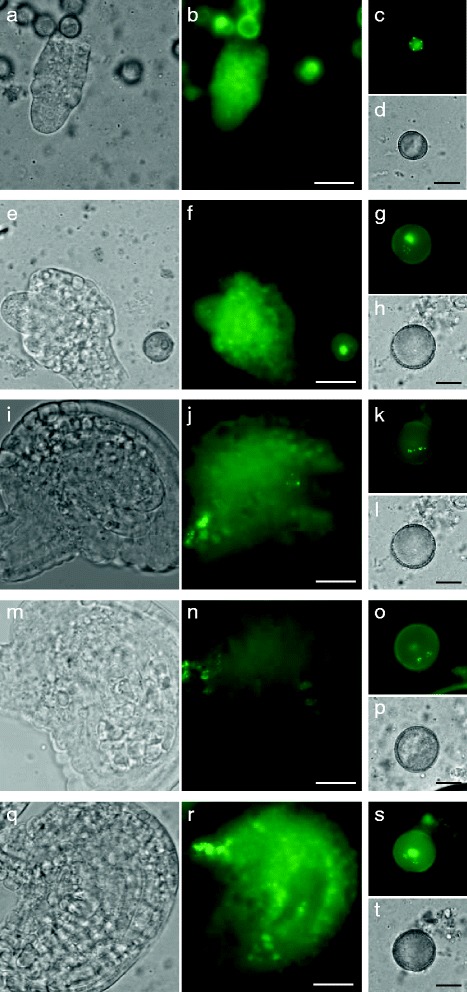
Gamete-specific centromere labeling in female *pWOX2-CENH3-GFP* embryo sacs. **a**-**t** Bright field imaging and GFP-based assessment of *pWOX2-CENH3-GFP* expression and localization during female reproduction; pre-meiotic stage **a** and **b**, meiosis **e** and **f**, uninuclear megaspore **i** and **j**, binuclear embryo sac **m** and **n** and fully matured seven-celled embryo sac **q** and **r**. Arrows indicate gametophytic cells. Scale bar, 20 μm. Corresponding stages in male gametophytic development are depicted next to each ovule figure; uninuclear **d** and **e**, binuclear microspore **g** and **h** and trinuclear **k**, **l**, **o**, **p**, **s** and **t** pollen stage. Scale bar, 10 μm. Images are single snapshots and hence not all centromeric signals may be displayed

In search for a gamete-specific centromere marker, we found that *pWOX2-CENH3-GFP* is expressed in both male and female gametogenesis and specifically localizes to the centromeres of gametophytic nuclei (Figs. 2 and 3). Moreover, in contrast to other lines that express *CENH3-GFP* in developing spores, *pWOX2*-driven expression is strictly confined to the gametophytic cell lineage, and is not observed in the enveloping tissues (e.g. ovule integuments), allowing a more accessible determination of the gametophytic ploidy level.

In male reproductive development, *pWOX2-CENH3-GFP* does not show any expression in meiosis or early gametogenesis (Fig. 2a and b). Starting from the late uninuclear microspore stage, however, *CENH3-GFP* is expressed and generally shows five fluorescent signals (Fig. 2d-f), corresponding to the haploid chromosome number in meiotically reduced spores. In the subsequent binuclear (Fig. 2g and h) and trinuclear stage, *pWOX2-CENH3-GFP* microspores also show labeling of centromeric regions with the constitutive presence of five fluorescent dots in the reproductive cell lineage (e.g., generative cell and sperm nuclei). In contrast, in the vegetative cell, CENH3-GFP does not show a dotted pattern but instead displays diffuse nuclear labeling (Fig. 2g). We therefore conclude that *pWOX2-CENH3-GFP* allows for the direct quantification of the absolute centromere number in the male generative cell lineage, but not in the vegetative cell.

It should be noted that the presence of multiple nuclei and the more condensed nature of the generative cells in both bi- and tri-nuclear spores often hamper accurate quantification of GFP signals (Fig. 3). The late uninuclear microspore stage, with its less-condensed nuclear configuration, is therefore the most optimal stage for CENH3-based male gametophytic chromosome quantification. At this stage, most spores (86.3 ± 7.6 %) display five distinct GFP signals, whereas only a minor set exhibits less than five fluorescent signals (12.7 % with 4 and 0.9 % with 3 GFP dots) (Fig. 2i). Although this suggests for aneuploidy, extensive analysis of male meiotic chromosome behavior revealed that *pWOX2::CENH3-GFP* plants do not show any MI or MII segregation defect and always produce haploid spores (*n* = 76) (Table 2), indicating that chromosome segregation imbalances do no occur or are extremely rare in diploid meiosis. In support of this, spores with more than five GFP dots were never observed (Fig. 2i). As a consequence, spores with three or four CENH3-GFP signals most likely represent stages of active centromere loading or correspond to events of centromere co-localization. In support of the latter hypothesis, we found that nuclei with less than 5 fluorescent dots often display one or two signals that are substantially larger or that show an enhanced fluorescence intensity (Additional file 5: Figure S4; see arrows).

**Table 2:** Gametophytic chromosome quantification using meiotic spreads and *pWOX2-CENH3-GFP*

Ploidy	Method	*n*		Microspore frequency (%)								
				# Chromosomes								
				1	2	3	4	5	6	7	8	9
Diploid	MII	72	Mean	0.0	0.0	0.0	0.0	100.0	0.0	0.0	0.0	0.0
			range	--	--	--	--	0.0	--	--	--	--
	CENH3	531	Mean	0.0	0.0	0.9	12.7	86.3	0.0	0.0	0.0	0.0
			SD	--	--	1.3	6.5	7.6	--	--	--	--
				# Chromosomes								
				6	7	8	9	10	11	12	13	14
Tetraploid	MII	66	Mean	0.0	0.4	2.7	9.9	78.8	8.0	0.4	0.0	0.0
			range	--	0.6	3.7	6.4	6.4	3.7	0.6	--	--
	CENH3	238	Mean	0.0	1.7	7.8	15.6	64.4	8.4	1.7	0.3	0.0
			SD	--	1.4	2.6	1.8	2.7	2.9	1.4	0.6	--
				# Chromosomes								
				3	4	5	6	7	8	9	10	11
Triploid	MII	52	Mean	0.0	1.1	5.4	13.1	30.5	30.5	13.1	5.4	1.1
			range	--	1.6	3.1	3.2	3.2	3.2	3.2	3.1	1.6
	CENH3	732	Mean	0.0	0.5	6.6	15.3	31.3	29.9	11.2	2.4	0.4
			SD	--	0.7	0.6	4.8	3.5	2.5	3.9	2.8	0.9

Distribution of the absolute number of chromosomes in male gametes resulting from MII meiotic chromosome segregation analysis and distribution of centromeric GFP dots in uninuclear microspores of diploid, triploid, and tetraploid Arabidopsis *pWOX2-CENH3-GFP* lines. For triploid meiosis, data from maternal and paternal excess plants was combined. In triploid and tetraploid meiosis, the occurrence of lagging chromosomes in MI (resulting in polyads; respectively 4.4 and 9 %) was integrated in the predicted ploidy distribution of the resulting gametes. As lagging chromosomes are not always detected in MII meiotic spreads (e.g. due to accidental polar localization by performing the chromosome spread), the frequency of polyad figures, obtained by or orcein-stained tetrad analysis, was used to determine the level of chromosome missegregation and forms the basis for the frequency ranges described

Besides male gametogenesis, *pWOX2-CENH3-GFP* is also expressed in female gametogenesis. Following meiosis, in which no fluorescent foci were observed (Fig. 3e and f), clear *CENH3-GFP* expression and associated centromere labeling was detected in generative cells during the whole process of megasporogenesis; from the uninuclear up till the seven-celled embryo sac stage (Fig. 3j, n and r). This gamete-specific centromere labeling was deduced from the position of the labeled nuclei in the embryo sac and was also confirmed by the constitutive presence of five centromeric dots in all GFP-expressing embryo sac nuclei (*n* = 57). Strikingly, in the surrounding tissue layers, GFP was not observed, except for a region in the funicle (Fig. 3j), indicating that *pWOX2-CENH3-GFP* allows for the unbiased detection and quantification of centromeres in female generative nuclei.

Similarly to male sporogenesis, quantification of CENH3-associated GFP signals in later stages of female gametogenesis (tetra- and eight-nuclear stage) is often hampered by autofluorescence of the enveloping tissue and progressive chromosome condensation of the reproductive nuclei (Fig. 3r). Optimal quantification of the female gametophytic chromosome number is therefore best performed at the uni- or binuclear stage (Fig. 3i, j, m and n). At this stage, female gametophytic nuclei consistently display five GFP spots (*n* = 57), typically reflecting the haploid genome dosage of meiotically reduced megaspores (Additional file 6: Figure S5).

### *pWOX2::CENH3-GFP* as a tool to analyze gametophytic ploidy and meiotic stability

Meiosis reduces the somatic chromosome number by half and generates gametes that all have the same haploid ploidy level. However, under certain circumstances, such as somatic polyploidy and meiotic defects, the meiotic outcome is altered and leads to poly- or aneuploid spores. To check whether *pWOX2-CENH3-GFP* can be used to detect and to quantify aberrations in male and female meiotic chromosome segregation, we assessed the absolute number of CENH3-GFP dots in spores resulting from triploid and tetraploid meiocytes.

**Fig. 4:**
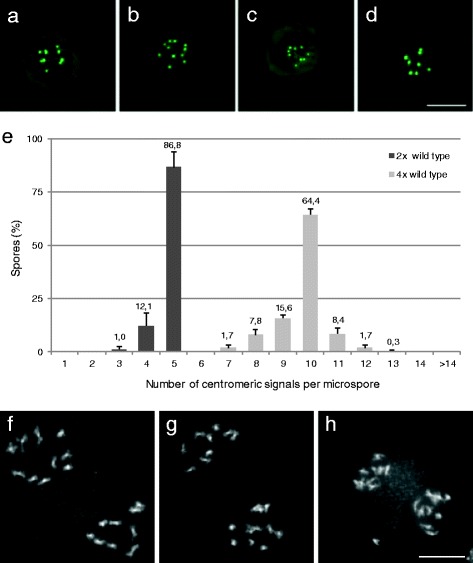
Male gametophytic ploidy distribution in tetraploid Arabidopsis using *pWOX2-CENH3-GFP*. **a**-**d** Late uninuclear microspores from tetraploid Arabidopsis expressing *pWOX2-CENH3-GFP*. Images are processed z-stacks. The microspores either contain the diploid number **a** or an aneuploid number of centromeric GFP signals **b**-**d**. **e** Frequency distribution of CENH3-GFP signals in nuclei of late uninuclear microspores isolated from diploid and tetraploid Arabidopsis controls. Error bars represent standard deviation (of three independent analyses of at least 100 spores). **f**-**h** Representative images of male meiotic chromosome spreads at the start of MII in a wild type tetraploid line showing either a balanced **f** or an unbalanced **g** and **h** segregation of homologous chromosomes in MI. In some cases, one or more lagging chromosomes are observed **h**. Scale bars, 10 μm

Tetraploid *pWOX2-CENH3-GFP* meiocytes were obtained by treating diploid seedlings with colchicine and by selecting tetraploid progeny plants. In a diploid *pWOX2-CENH3-GFP* line, uninuclear microspores predominantly exhibit the haploid number of 5 centromeric dots (86.3 %) and occasionally show a lower number of fluorescent foci (13.6 %). In contrast, microspores from a tetraploid *pWOX2-CENH3-GFP* line show much more variability in the number of GFP dots, ranging from 7 up till 12 (Fig. 4a-d). The majority of these nuclei show ten GFP signals, representing the haploid ploidy level in a tetraploid background (4x = 20; Fig. 4e). Spores with less than the expected number of GFP dots (<10) may result from the co-localization of two or more chromocenters (Fig. 4a), similarly as observed in diploids. In contrast, microspores with more than the expected number of GFP signals were never observed in diploids, indicating that their presence in the tetraploid background evidences gametophytic aneuploidy (Fig. 4b), most likely caused by defects in meiotic chromosome segregation. To validate this, we analyzed meiotic chromosome segregation in diploid and tetraploid PMCs and compared this with the gametophytic ploidy distribution obtained by *pWOX2-CENH3-GFP*. In the diploid background, male meiosis always generates balanced tetrads and yields haploid spores with five (x = 5) chromosomes (Table 2). Similarly, in *de novo* tetraploids, most PMCs generally perform a balanced meiosis (Fig. 4f), yielding diploid (2x = 10) spores. However, due to MI tetravalency, some tetraploid PMCs exhibit aberrations in MI chromosome segregation and consequently generate aneuploid spores. Male meiotic chromosome spreading revealed that 16.7 % of neo-tetraploid male meiocytes undergo unbalanced MI chromosome segregation, typically resulting in a 9–11 (15.2 %) or a 8–12 pattern (1.5 %) at the start of MII (Fig. 4g). Interestingly, male meiotic analysis in neo-tetraploids did not only reveal tetrads but also showed some polyads (9 %; *n* = 255), which typically contain one or two micronuclei. Polyad presence indicates for the occurrence of MI chromosome lagging or defects in meiotic chromosome segregation (Fig. 4h). As a result, in neo-tetraploid *Arabidopsis thaliana* plants, the actual number of microspores with a specific amount of chromosomes is variable and is expected to range between values outlined in Table 2.

Comparative ploidy analysis in tetraploid male gametogenesis demonstrates that the gametophytic ploidy distribution obtained by *pWOX2-CENH3-GFP* centromere labeling closely corresponds to the actual meiotic chromosome segregation pattern (obtained by chromosome spreads). The bias of CENH3 signal distribution to spores having less than ten centromeric dots (25.1 % vs. expected 16.55 %) is most likely caused by the interference of lagging chromosomes and by the occasional co-localization of two or more centromeric regions.

**Fig. 5:**
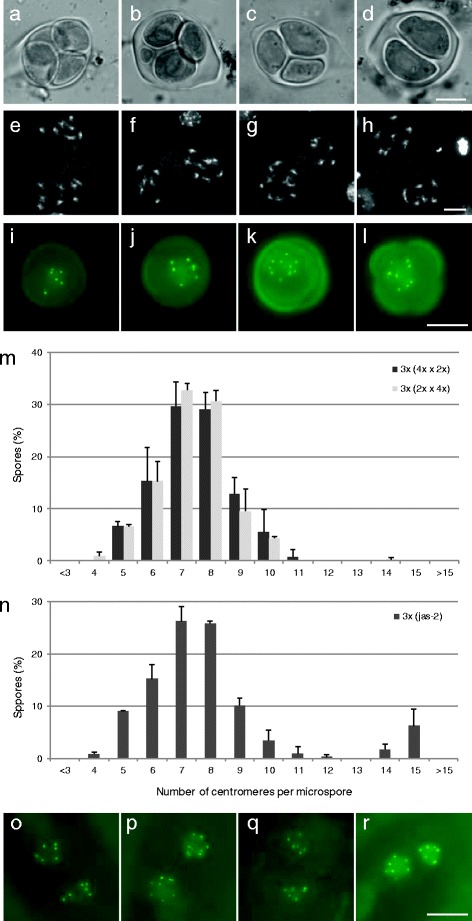
Gametophytic ploidy distribution using *pWOX2-CENH3-GFP* in triploid Arabidopsis. **a**-**d** Representative images of male meiotic outcome in wild type (**a**, unbalanced tetrad; **b**, polyad) and *jason-2* (**c**, triad; **d**, dyad) triploid lines. **e**-**h** DAPI-stained chromosome spreads of male meiocytes at the beginning of MII in triploid Arabidopsis shows lagging chromosomes **h** and an unbalanced segregation of homologous chromosomes. **i**-**l** GFP fluorescence images of late uninuclear microspores isolated from a triploid *pWOX2-CENH3-GFP* line. Images are processed z-stacks. Scale bars, 10 μm. **m**-**n** Frequency distribution of pWOX2-CENH3-GFP signals in nuclei of late uninuclear microspores isolated from triploid Arabidopsis control lines **m** and a male overdose triploid *jason* line **n**. Error bars represent standard deviation (of three independent analyses of at least 100 spores). **o**-**r** Fluorescent imaging of binuclear megaspores in developing ovules of triploid *pWOX2-CENH3-GFP*. Scale bars, 10 μm

In addition to tetraploids, we also created triploid *pWOX2-CENH3-GFP* lines by performing reciprocal 2x-4x crosses. Moreover, to further validate whether *pWOX2-CENH3-GFP* can be used to detect alterations in meiotic chromosome segregation, we also assessed a triploid *jason* mutant. Due to a defect in MII spindle orientation, *jason* male meiosis yields triads and dyads that contain unreduced gametes (Fig. 5c-d) [9, 48]. As a result, triploid *jason* is expected to generate a subset of triploid male gametes.

In triploid wild type plants, PMCs always form five trivalents instead of five bivalents at MI (*n* = 22) (Additional file 7: Figure S6). In anaphase I, these trivalents segregate one chromosome to one pole and two to the other pole. Consistent with an expected random chromosome segregation of each individual trivalent, triploid meiosis I predominantly results in a 7–8 distribution (60.9 %), and to a lesser extent in a 6–9 (26.1 %) or 5–10 (13.0 %) distribution (Fig. 5e-g). Moreover, although triploid PMCs predominantly yield tetrads (Fig. 5a), they also occasionally form polyad structures (4.4 %; Fig. 5b); e.g. tetrads with extra micronuclei, indicating for the occurrence of MI chromosome lagging (Fig. 5h). Considering these alterations, the expected ploidy distribution of spores generated by triploid PMCs varies according to the values presented in Table 2. In line with this, we found that the number of CENH3-GFP dots in spores produced by triploid PMCs shows a Gaussian distribution with most spores containing 7 or 8 fluorescent dots and a smaller set of spores displaying less or more GFP signals (Fig. 5i-l). Both types of triploids (e.g. resulting from reciprocal *2n* x *n* crosses) showed a similar CENH3-GFP signal distribution at the uninuclear microspore stage (Fig. 5m), indicating that the parental genome dosage in the triploid does not affect the pattern of meiotic chromosomal segregation. Moreover, considering both types of triploids, we found that the distribution of CENH3 signals in uninuclear microspores closely corresponds to the actual meiotic chromosome segregation numbers (Table 2). Indeed, the frequency of microspores with a specific number of CENH3-GFP signals (4–11) always amounts within the range of values obtained from the MII meiotic chromosome analysis.

Similarly, in triploid *jason*, the number of CENH3-GFP signals at the uninuclear microspore stage showed a Gaussian distribution and ranged from 4 to 12 with most spores containing 7 or 8 centromeric dots (Fig. 5n). In addition, triploid *jason* also produces a subset of spores with 14 or 15 GFP signals (1.72 and 6.26 % respectively). As the somatic chromosome number in Arabidopsis triploids equals fifteen, these spores seemingly have a somatic rather than a gametophytic chromosome number. This is in line with the functional loss of JASON, which causes meiotic non-reduction and the associated production of unreduced gametes. Spores with 14 CENH3-GFP signals are most likely unreduced gametes with 15 chromosomes, in which one CENH3 signal is not detected due to centromere co-localization (physical clustering or through image-based z-stacking).

Based on these findings, we conclude that the in vivo localization of CENH3 in late uninuclear microspores is a valuable tool to assess the stability of male meiotic chromosome segregation and to detect potential alterations in male gametophytic ploidy, such as aneuploidy and 2n gametes.

To assess whether *pWOX2-CENH3-GFP* also enables accurate chromosome quantification in female gametogenesis, we monitored the absolute number of GFP signals in the two-celled embryo sac stage of triploid plants. For both types of triploids (maternal and paternal excess), the number of GFP signals in the female gametophyte showed a Gaussian-like distribution with values ranging from 4 to 10 (Fig. 5o-r). However, in contrast to the male germline, in which most cells contained the expected 7 or 8 centromeric signals, the majority of the embryo sac nuclei only contained 6 or 7 GFP dots. The other cells exhibited either less (4–5) or more centromeric GFP signals (8–10) (Additional file 8: Figure S7). This distribution pattern deviates from the expected gametophytic ploidy distribution, as calculated from a random segregation of homologous chromosomes in triploid meiosis, and most likely is caused by a selective elimination of megaspores or CENH3 co-localization.

### *pWOX2-CENH3-GFP* allows early embryonic ploidy determination

**Fig. 6:**
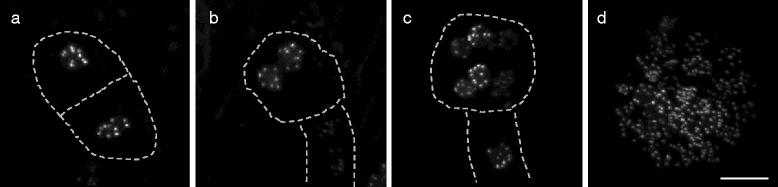
CENH3-GFP centromere labeling allows ploidy determination during early embryogenesis. **a**-**d** Centromere-specific labeling of the recombinant pWOX2-CENH3-GFP protein in early stages of embryo and zygote development: one-celled stage **a**, four-celled stage **b**, eight-celled stage **c** and the late globular embryo stage **d**. Nuclei always show ten centromeric GFP dots. Scale bar, 20 μm

Expression studies in Arabidopsis have demonstrated that the homeobox protein WOX2 is not only transcribed in the female gametophyte (e.g. egg cell), but also in the zygote where it is confined to the apical meristem [49, 50]. Based on this expression profile, we monitored *pWOX2-CENH3-GFP* expression in early embryo development and found a similar labeling of centromeric regions in all cells examined. Indeed, in the early bi-, tetra- and eight-cellular embryo stage, ten fluorescent foci were observed in all embryonic nuclei (Fig. 6a-d), clearly reflecting a centromere-specific labeling. In addition, we noticed that *pWOX2-CENH3-GFP*-mediated centromere labeling is not only confined to the apical region, but is also present in the basal nuclei (Fig. 6c). In line with this, we occasionally observed globular-staged *pWOX2-CENH3-GFP* embryos (>32 nuclei) in which all nuclei (e.g., basal, central and apical) display ten fluorescent dots (Fig. 6d). Hence, *pWOX2-CENH3-GFP* also labels centromeric regions in developing embryos and thus provides an easy tool to assess the basic ploidy level (e.g., di-, poly- or aneuploid) at the earliest stage of plant development.

### CENH3-GFP as a marker for somatic ploidy determination

**Fig. 7:**
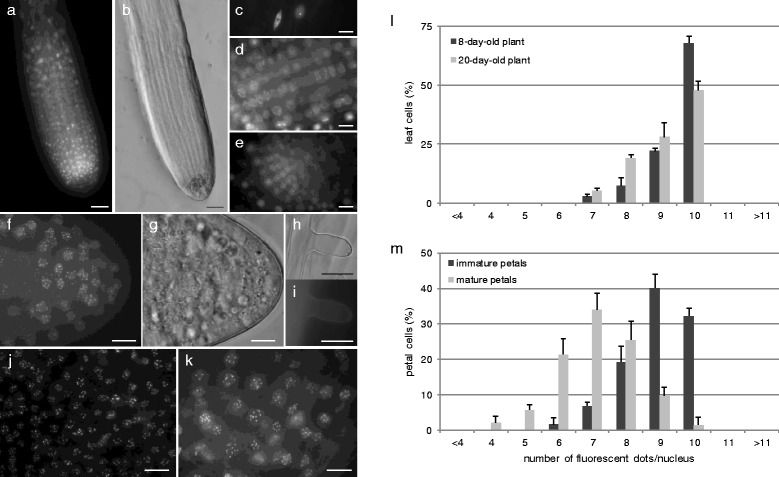
Characterization of *p35S-CENH3-GFP* expression in Arabidopsis plant development. **a**-**g** Expression profile and centromere-specific localization of the p35S-CENH3-GFP recombinant protein in the primary root tip **a** and **b**, root elongation zone **c**, root differentiation zone **d**, the proliferating root tip **e** and the lateral root tip **f** and **g. a**-**b** Scale bars, 25 μm. **c**-**g** Scale bars, 10 μm. **h**-**i** Bright field and GFP fluorescence of a *p35S-CENH3-GFP* root hair. Scale bar, 20 μm. **j**-**k** Centromere-specific p35S-CENH3-GFP signals in emerging leaves of 8-day-old seedlings **j** and mature petals at anthesis **k**. Scale bars, 10 μm. **l**-**m** Frequency distribution of centromeric GFP signals in nuclei of young leaves **l** and petals **m** at different stages in development. Error bars represent standard deviation (of three independent analyses of at least 100 spores)

In contrast to most CENH3-GFP constructs tested, which only show expression in one or some tissues, the constitutive *p35S*-*CENH3-GFP* is expressed in a large set of organs throughout plant development. In seedlings and young plants, *p35S*-*CENH3-GFP* expression has been observed both in roots (Fig. 7a-g) and leaves (Fig. 7j), but not in root hairs and trichomes (Fig. 7i). In later stages of development, most plant organs - including roots, leaves and reproductive structures (petals, sepals) - exhibit *CENH3-GFP* expression and thereby show a dotted pattern, indicating for centromere-specific labeling (Fig. 7k and 1o-r). A similar expression pattern was obtained by the *CENH3 GFP-tailswap* reporter driven by the endogenous *CENH3* promoter (Additional file 9: Figure S8), indicating that *p35S-CENH3-GFP* enables centromere labeling in all cell types that show endogenous expression and centromeric incorporation of CENH3.

Based on its broad expression in different plant organs, we hypothesized that *p35S-CENH3-GFP* allows for the in vivo quantification of the absolute number of chromosomes in different somatic cell types. To validate this, we performed a broad-scale fluorescent foci quantification assay in diploid and tetraploid *p35S-CENH3-GFP* plants covering several types of plant organs.

In young leaves of eight- and 20-day-old diploid Arabidopsis *p35S-CENH3-GFP* plants, we found that the majority of cells (respectively 67.9 ± 2.8 % and 47.8 ± 3.9 %) display exactly ten fluorescent dots in the nucleus (Fig. 7l). As this number corresponds to the absolute number of centromeres in diploid cells, we conclude that the p35S-CENH3-GFP protein labels all chromocenters and hence can be used to determine the ploidy level of single cells. However, in both types of leaves, we additionally found a subset of cells (32.1 and 52.2 % respectively) with less than ten dots (Fig. 7l). These cells generally contain one or two dots that are substantially larger with an enhanced fluorescence intensity (Fig. 7j, arrows), indicating that they do not result from biological aneuploidy, but rather from the in vivo co-localization of centromeric regions. In support of this, cells with more than ten CENH3-GFP foci were never observed (Fig. 7l). Strikingly, in 20-day-old plants, the frequency of cells showing the diploid number of ten centromeric foci was significantly lower compared to eight-day old plants, whereas the number of cells with less than ten CENH3 dots was substantially larger (Fig. 7l). From this we conclude that, in the perspective of in vivo somatic ploidy determination, CENH3-GFP-mediated chromosome quantification is optimally performed during early stages of plant development.

In immature petals, assessed at flowering stage 5–6, a similar distribution of *p35S-CENH3-GFP* foci was observed as in the leaves (Fig. 7k). However, in contrast to the prevalent presence of ten dots in emerging leaves, immature petals showed a slight reduction in GFP dot frequency, with the majority of cells displaying 10 or 9 dots and all other cells showing a reduced number of 6, 7 or 8 dots (Fig. 7m). Nuclei with more than ten GFP dots were not observed. At the end of the reproductive phase, in fully matured flowers, p35S-CENH3-GFP still shows a centromere-specific localization pattern. However, in contrast to seedlings and young petals, nuclei in fully matured petals do not predominantly contain 9–10 GFP dots but instead display a severe reduction in the number of centromeric dots (Fig. 7m). More specifically, in mature petals, nuclei predominantly contain 6, 7 or 8 GFP signals whereas only a small subset shows a lower (e.g., 4 and 5) or a higher number (e.g., 9 and 10). Cells with more than ten GFP signals were again not observed. Cells with less than ten GFP dots consistently showed one or more enlarged fluorescent signals, indicating that the decrease in GFP signals was not caused by chromosome or centromere loss, but rather by the in vivo co-localization of centromeres. In support of this, flow cytometric DNA analysis showed that the relative genomic DNA content of mature petal nuclei did not deviate from nuclei isolated out of young immature flower buds or twenty-day-old seedling leaves (Additional file 10: Figure S9).

### CENH3-GFP allows for the detection and characterization of endomitotic polyploidy

In polyploid cells, discrimination between endomitosis and endoreduplication cannot be established using DNA flow cytometry, but instead can be assessed cytologically, e.g. by determining the absolute chromosome number. To check whether CENH3-GFP-mediated centromere labeling can be used to differentiate between these two types of polyploid cells, we performed two experiments in which endomitotic polyploidy was ectopically induced in the *p35S-CENH3-GFP* background. In the first experiment, seven-day-old seedlings were treated with the mitotic blocking agent colchicine. In the second experiment, *p35S-CENH3-GFP* was introduced in the Arabidopsis mutant *et2*, which has a mutation in *GSL8* (GLUCAN SYNTHASE-LIKE 8; a callose synthase required for cell wall formation) and consequently displays ectopic endomitotic polyploidy events through defects in cell wall formation [51].

**Fig. 8:**
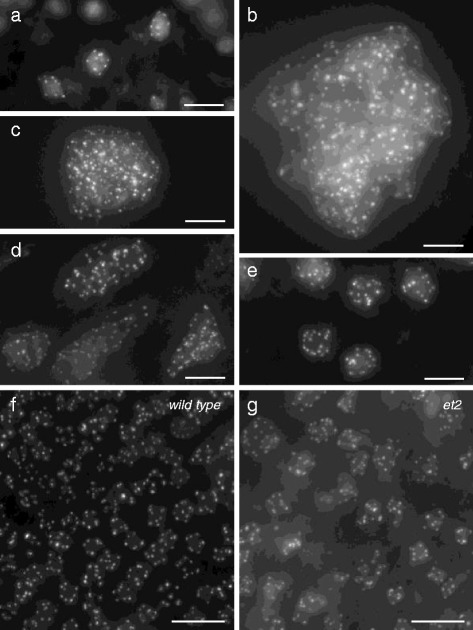
Somatic *p35S-CENH3-GFP* centromere labeling detects endomitotic polyploidy. **a**-**e** Representative images of somatic nuclei expressing *p35S-CENH3-GFP* in young leaves of wild type **a** and colchicine-treated seedlings at seven days **b** and **c** and twenty days **d** and **e** after treatment. **f** and **g** Fluorescent images of p35S-CENH3-GFP localization in young developing petals in diploid wild type **f** and *et2* **g** background. Note the ectopic presence of large endomitotic nuclei containing more than ten centromeric GFP signals in *et2* petals. Scale bars, 10 μm

In the colchicine induction experiment, seedlings showed a significant retardation of leaf growth and development during the first days post treatment (dpt). Starting from day five, plantlets that recovered the toxic treatment initiated organ development and formed new leaf initials. To check whether CENH3-GFP allows for the detection of colchicine-induced endomitotic polyploidy, nuclei in these newly formed organs were assessed using fluorescence microscopy. In contrast to somatic cells in non-treated plantlets, which typically contain seven to ten centromeric GFP signals (Fig. 8a), nuclei in emerging leaves of colchicine-treated plants at 7 dpt were significantly larger and displayed a substantial increase in centromeric GFP signals (Fig. 8b and c). In most nuclei the total number of fluorescent signals was too high (exceeding 150) to quantify the exact number of chromocenters. As some of these so-called ‘hyper-polyploid’ nuclei showed an amorphous bubble-like morphology, with multiple individual nuclei clustered together in one nuclear body structure (Fig. 8b), we conclude that these nuclei originate from the repeated loss of cell wall formation and the associated fusion of syncytial nuclei. Strikingly, at later stages upon colchicine treatment (20 dpt), a strong reduction in hyper-polyploid nuclei was observed and nuclei in newly formed leaves only showed a moderate increase in GFP foci number (e.g. ranging from 50 to 100) (Fig. 8d). Moreover, in some plants, nuclei of young emerging leaves again appeared uniformly shaped and only contained 15–23 fluorescent dots (Fig. 8e), indicating that the ploidy level of the meristematic region has been duplicated, thereby putatively establishing a stable tetraploid basic ploidy number.

Endomitotic cells in the Arabidopsis *et2* mutant are ectopically formed by the occasional alteration of cell wall formation and subsequent fusion of syncytial nuclei [51]. Although the induction of *et2* endomitosis is generally low, some organs and particularly flower tissues (e.g., petals) are more sensitive and show a higher incidence of endomitotic polyploidy. To check whether *p35S-CENH3-GFP* can be used to detect and to quantify endomitotic cells, we crossed the CENH3-GFP reporter construct in the *et2* background and assayed GFP fluorescence in young petals. In contrast to the uniformly sized nuclei in wild type petals, which typically contain ten or less centromeric dots, *et2* petals exhibited enlarged nuclei that clearly display more than ten dots (Fig. 8f and g). In these nuclei, the total number of CENH3-GFP signals ranges from 16 to 20, closely corresponding to the number of centromeres in tetraploid nuclei. Moreover, in some *et2* petal cells more than 20 GFP signals were observed, indicating that besides tetraploid cells also cells with a higher ploidy level are formed. Similarly, in other organs that normally do not show endopolyploidy (e.g., sepals, stamen filaments), p35S-CENH3-GFP-mediated centromere labeling also revealed enlarged nuclei with an increased number of GFP foci, strongly reflecting the ectopic presence of endomitotic polyploidy in several *et2* organ types.

Based on these findings, we postulate that the in vivo labeling of centromeric proteins, such as CENH3, provides an easy methodology to detect and to characterize endomitotic polyploidization events.

## Discussion

In this study we present the use of recombinant CENH3-GFP reporter constructs as a tool to quantify the number of chromosomes in both somatic and gametophytic cell types. Based on a reproduction-specific reporter screen, constituted by modulating CENH3-GFP expression through different promoters, we isolated two reporter lines that enable clear centromere labeling in exclusively gametophytic (*pWOX2-CENH3-GFP*) and somatic (*p35S-CENH3-GFP*) tissues types. Based on these lines, we demonstrate that recombinant CENH3-GFP allows an in vivo chromosome quantification in different cell and tissue types and hence provides an easy and straightforward method to determine the somatic ploidy level and gametophytic ploidy distribution in Arabidopsis male and female gametogenesis.

In plants, like in all eukaryotic organisms, CENH3 is a centromere-specific protein that functions as a constitutive substitute of histone H3 within the nucleosomes of active centromeres [33]. In Arabidopsis, the centromere-specific localization of CENH3 has already been demonstrated using both recombinant and immunocytological approaches [33, 47, 52, 53]. However, in none of these reports, the use of recombinant CENH3 as an in vivo marker for somatic and gametophytic ploidy analysis has been described. Here, in this study, we confirm that ectopically expressed *CENH3-GFP*, under the control of different promoters, specifically localizes to the nuclear chromocenters in a large variety of plant organs, including developing male and female gametes. Indeed, in all CENH3 reporter lines studied, except for *pLAT52-CENH3-GFP*, GFP fluorescence showed a nuclear localization pattern and exhibited the expected number of ten (or less) and five centromeric dots in somatic and gametophytic cell types, respectively. Ectopic expression of CENH3-GFP does not confer any cytoplasmic or non-nuclear localization, indicating that the expression of the recombinant reporter in the selected lines does not exceed the physiological threshold level and thus enables a distinct labeling of centromeric chromosome regions. Moreover, since all CENH3-GFP harboring plants do not show any physiological or developmental defect and display a growing behavior similar to wild type, we conclude that the introgression of recombinant CENH3 proteins, in addition to the endogenous CENH3 protein, does not interfere with the functional role of the centromere in mitotic and meiotic cell division. In contrast, in a recent study by Lermontova et al. (2011), the expression of a N-terminally truncated EYFP-CENH3 construct has been found to strongly reduce endogenous CENH3 mRNA transcript levels and to severely affect meiotic chromosome segregation and seed set [54]. Similar meiotic defects and associated sterility have also been observed in Arabidopsis *cenh3* mutants that express a GFP-tagged chimeric CENH3 protein consisting of an H3 N-terminal tail combined with the normal CENH3 C-terminus (GFP-tailswap) [52]. In our transgenic CENH3-GFP lines, both the formation of male and female gametes and subsequent seed generation were not affected, indicating that the introgression of a recombinant CENH3-GFP protein does not severely alter endogenous CENH3 levels and hence does not interfere with somatic and reproductive cell divisions.

In search for a marker that allows for the in vivo detection of centromeres in Arabidopsis sporogenesis, we found that expression of CENH3-GFP under the control of several meiosis- and gametophyte-specific promoters does not allow for a distinct labeling of centromeres during male meiotic cell division. In contrast, GFP expression in these lines was observed in somatic tissues, indicating that the absence of CENH3 labeling in male meiosis is not due to irregularities in cloning or construct design, but instead is attributed to other factors, such as epigenetic silencing (e.g. enhanced histone folding or methylation) or protein-related defects in CENH3-GFP loading. In a recent report, Ravi et al. (2011) demonstrated that alterations in the N-terminal tail of the CENH3 protein specifically impair centromeric loading in meiotic cells, but not in mitotic cells, indicating for the existence of a specialized meiotic CENH3 loading pathway [52]. As such, alterations in CENH3 protein constitution (e.g., by GFP fusion) may potentially hinder CENH3 loading in meiosis I and II. Opposed to this hypothesis, Ravi et al. (2011) demonstrated that the ectopic expression of a recombinant GFP-CENH3 marker protein enables clear labeling of centromeric regions in all stages of meiosis. However, as this was obtained in a mutant *cenh3-1* background, which causes a complete lack of CENH3 at the kinetochore, proper GFP-based CENH3 labeling in meiosis may require the absence of the native CENH3 protein. It should also be noted that meiotic GFP-CENH3 visualization in the study of Ravi et al. (2011) was obtained by using a glycerol-based staining buffer, indicating that the type of buffer may also affect fluorescent GFP image acquisition. Visualization experiments with the same buffer did not reveal meiotic GFP labeling in our CENH3-GFP lines, suggesting that the absence of meiotic labeling is most likely caused by epigenetic silencing or by a reduced, non-detectable expression of the inserted construct in the meiotic phase.

The fluorescent screening experiment additionally revealed that Arabidopsis wild type PMCs show one or more fluorescent dot in all stages of meiosis, confounding the analysis of dotted GFP fluorescence patterns. The autofluorescence is not only confined to the Colombia-0 ecotype but also occurs in several other ecotypes (data not shown), suggesting that it is a common property of *Arabidopsis thaliana* meiocytes. Although never documented as autofluorescence, some research groups examining meiosis-specific protein expression have reported similar meiotic GFP profiles [55, 56]. To analyze to what extent these reported GFP signals represent genuine protein-specific localization patterns, or rather constitute meiotic autofluorescence as observed in our study, additional control experiments may be required. In any case, the presence of this meiotic autofluorescent signal may interfere with GFP-based fluorescent protein acquisition, putatively impairing the use of specific GFP-based reporters, such as CENH3-GFP, in Arabidopsis male meiosis. However, as the autofluorescence only appears up till the early microspore stage, CENH3-GFP-mediated centromere labeling in subsequent gametogenesis stages is not hampered, providing a developmental window in which CENH3-GFP allows for male gametophytic chromosome quantification and ploidy determination. Moreover, as the frequency of centromeric GFP signals in uninuclear microspores closely correlates to the segregation of chromosomes in both diploid, triploid and tetraploid PMCs, we here postulate that the *pWOX2-CENH3-GFP* reporter provides an excellent tool to determine the ploidy distribution of developing male gametes in a quick, non-destructive manner.

Strikingly, in female spores generated by a triploid meiosis, CENH3-GFP-mediated centromere quantification did not show the expected Gaussian distribution as observed in male meiosis, but instead exhibited a significant shift to a lower number of centromeric signals (e.g., 6–7 dots). Since this distribution deviates from the expected ploidy distribution randomly generated by triploid meiosis, we postulate that female sporogenesis shows a bias towards gametes that have a lower number of chromosomes. This could be caused by the selective degeneration of higher ploidy gametes in the post-meiotic uninuclear megaspore stage or may be attributed to the clustering of centromeric regions in aneuploid or polyploid gametes. As diploid *pWOX2-CENH3-GFP* plants consistently show five CENH3-GFP dots in the nuclei of young megaspores, clustering of centromeric regions is thought to constitute only a minor component leading to a reduced number of centromeric signals.

The potential use of CENH3-GFP as a tool to assess the basic ploidy level of somatic tissue was assessed using the *p35S-CENH3-GFP* fusion construct. *CENH3-GFP* expression under the control of *p35S* results in centromere-specific GFP signals in a large number of organ types; including root tips, leaf meristems, young leaf material, flower meristems and developing flower organs (e.g. stamen, pistil, petal and sepals). Strikingly, no expression was observed in male and female reproductive cells or in developing meiocytes. Although this is in line with the generally held view that the CaMV 35S promoter is virtually silent in reproductive tissues, our observation is in sharp contrast with previous studies in strawberry [57], tomato and tobacco [58, 59], in which p35S appeared to drive expression of GUS in pollen and other reproductive tissues. Based on these and other results, Dutt et al. (2014) concluded that the transgene expression profile conferred by the CaMV 35S promoter is species-dependent and may show variability due to its interaction with environmental factors and the physiological state of the plant [60].

In vivo quantification of centromeric GFP signals in somatic tissue types (e.g. leaves and petals) not only revealed nuclei with the diploid number of 10 dots, but also displayed a substantial number of cells with a lower number of centromeric signals. A similar variability in centromeric GFP signals in diploid nuclei (7–10 signals) has also been reported by Lermontova et al. (2006) [33]. These authors additionally found that tetraploid and polyploid endoreduplicated nuclei often contain more than ten centromeric GFP signals, for example ranging up till 13 in octaploid nuclei. In contrast, in *p35S-CENH3-GFP* somatic tissue types, cells with more than 10 fluorescent signals were never observed, suggesting that the *p35S*-driven construct is not expressed in endoreduplicated cells or does not allow proper loading of CENH3-GFP in endoreduplicated cells. At the other hand, several cells with less than 10 GFP foci signals were observed in the *p35S-CENH3-GFP* line, suggesting for putative events of somatic aneuploidy. However, our study revealed that these nuclei do not originate from mitotic chromosome segregation instabilities, but instead result from the in vivo co-localization of one or more centromeric regions, indicating that several cells need to be considered to obtain an accurate quantification of the somatic ploidy level. It is thereby also important to note that both the developmental age and physiological stage have an additional effect on the pattern of CENH3 centromeric loading. Indeed, in several somatic tissues and particularly in petals, the frequency of centromeric GFP signals in individual nuclei was not stable upon development but instead shifted to a lower number. Since DNA flow cytometry hereby did not reveal accumulation of aneuploidy and cells with more than ten GFP signals were not observed, the reduced number of CENH3 foci is most likely caused by a progressive clustering of centromeric regions during organ development. In yeast and many other eukaryotes, co-localization of centromeres is typically observed at interphase, showing a tight clustering at the peripheral side of the nucleus [61, 62]. This process is termed Rab1-orientation of chromosomes [63]. Studies in yeast have demonstrated that this interphase-specific centromeric clustering decreases in non-dividing cell types, suggesting that dividing tissues should show a more accurate labeling of centromeric regions later in development. In contrast, in our study, we observe a reduced number of CENH3-GPF dots in later developmental stages of Arabidopsis somatic tissues, putatively reflecting an increased number of interphase-arrested cells. Alternatively, the reduced number of CENH3-GPF dots in later developmental may be caused by an increasing number of endoreduplicated cells, which may exhibit an enhanced incidence of centromere co-localization [33]. However, since little is known about centromere dynamics in plants, more studies are needed to explain the progressive reduction in CENH3-GFP signals.

Due to the progressive centromere co-localization during Arabidopsis plant development, centromere number becomes more variable and deviates strongly from the actual ploidy level, indicating that CENH3-GFP cannot be used to quantify the absolute chromosome number of individual cells in mature leaf or petal tissues. But, as the number of centromeric dots never exceeds the actual number of somatic chromosomes, we postulate that *p35S-CENH3-GFP* can be used to determine the basic ploidy level, particularly in newly formed tissue types. We additionally found that *p35S-CENH3-GFP* allows for the detection and localization of ectopic endomitotic polyploidization events, for example induced by a mitotic inhibitor drug (e.g. colchicine). Indeed, as the genome duplication process of endomitosis results in an absolute doubling of chromosomes, whereas this is not the case for endoreduplication (polytenal chromosomes) [64, 65], in vivo quantification of the absolute centromere number offers an ideal methodology to discriminate between both types of endoploidy and provides data on the localization and severity of endomitotic polyploidization events.

## Conclusion

In this report we present the use of recombinant CENH3-GFP proteins as an easy and straightforward method to determine the cell-specific ploidy level (e.g. number of chromosomes) in several somatic and gametophytic Arabidopsis cell types.

Based on the analysis of CENH3-GPF reporter constructs under the transcriptional control of different promotor types, we found that *pWOX2::CENH3-GFP* specifically labels centromeres in gametes without the need of fixation or any other pretreatment of the cells. The marker allows in vivo quantification of the gametophytic chromosome number in both micro- and megasporogenesis. This has been validated by using both polyploid and 2n gamete producing lines and by comparing CENH3-based data with cytological chromosome distribution assessments. As such, we demonstrate that *pWOX2::CENH3-GFP* allows for the detection of rare events of meiotic aneuploidy or gametophytic polyploidy.

The analysis of a somatically expressed CENH3-GFP variant (*p35S::CENH3-GFP*) revealed centromeric labeling in a large set of cell types; including root tips, leaves and flower structures, thereby providing an efficient tool to determine the somatic chromosome number on a single cell basis. Analysis of different tissue types, however, revealed a progressive clustering of centromeric regions upon plant development and maturation, indicating that CENH3-GFP-based ploidy determination is optimally performed in young, emerging organ types. Moreover, using both colchicine- and mutant-based endoploidy induction, we demonstrate that recombinant CENH3-GFP provides an excellent method to detect and to characterize ectopic events of endomitotic polyploidization.

## Methods

### Plant material

Diploid and tetraploid Arabidopsis (Colombia-0 background) wild type accessions were obtained from the Nottingham Arabidopsis Stock Center (NASC). CENH3-GFP constructs were always generated in a diploid Colombia-0 (Col-0) background. Triploid lines were generated by performing manual reciprocal crosses between diploid and tetraploid controls. The Arabidopsis mutants *jason* and *et2* (for *enlargedtetrad 2*) were isolated out of an EMS mutagenized M2 Arabidopsis Col-0 seed stock (Lehle Seeds) as described previously [9].

Following an initial in vitro seed germination (6–8 days, K1 medium), Arabidopsis seedlings were cultivated in fully controlled climate chambers under the following conditions: photoperiod 12 h day/12 h night, temperature 20 °C and humidity <70 %. At the start of flower initiation, photoperiod settings were changed to 16 h day/ 8 h night under the same conditions to stimulate flowering. For analysis of seedling GFP expression, plantlets were kept on the plate in the growing chamber.

### Generation of reporter constructs

The CENH3-GFP reporter cassette driven by the different promotor sequences was constructed using the Multisite-Gateway cloning system according to manufacturer’s instructions (Invitrogen). Sequences of the primers used to clone the corresponding cDNA fragments are listed in Additional file 11: Table S2. Verified plasmids was transformed into the *Agrobacterium tumefaciens* strain GV3130 and used to generate transgenic Arabidopsis lines (ecotype Colombia-0) through floral dip methodology. Single T-DNA transgenic progeny plants were selected based on kanamycin resistance and stable GFP expression.

### Cytology and histology

Chromosome behavior during male meiosis was visualized cytologically using the well-established spreading technique [66] with some minor modifications [9]. After fixation of meiotic buds in 3:1 ethanol:acetic acid, buds were rinsed twice (1x distilled H_2_0 and 1X citrate buffer) and incubated in an enzyme mixture consisting of 0.3 % (w/v) pectolyase (Sigma) and 0.3 % (w/v) cellulase (Sigma) in citrate buffer at 37 °C in a moisture chamber for 1.5 h. Digested buds were subsequently rinsed and stored at 4 °C in citrate buffer. A single enzyme digested bud was transferred to a slide, macerated with a needle in a small drop of 60 % acetic acid and stirred gently on a hotplate at 45 °C for 30 s. The slide was then flooded with freshly made ice-cold 3:1 ethanol:acetic acid and subsequently air dried. Finally, slides were stained by adding 25 μl of DAPI (1 μg/ml) in Vectashield antifade mounting medium, mounted with a coverslip and squashed between filter paper to remove excess stain and mounting medium.

Analysis of the male meiotic outcome was performed by selecting meiotic buds based on size and shape (flowering stage 9) and squashing them on a slide in a drop of 4.5 % (w/v) lactopropionic orcein (LPO, Sigma-Aldrich) staining solution. Buds producing significant numbers of fully developed meiotic products (e.g. tetrad stage) were used for phenotypic analysis.

### Microscopy

Bright field and fluorescence imaging was performed using an Olympus IX81 inverted fluorescence microscope equipped with an X-Cite Series 120Q UV lamp and an XM10 camera. Confocal 3D imaging of CENH3-GFP localization was performed using a Nikon A1r laser scanning microscope equipped with Axiovision software (LiMiD). Image processing and z-stack projections were conducted using ImageJ.

### DNA Flow cytometry

The somatic ploidy level was determined using DNA flow cytometry (Epics Altra, Beckman) based on the nuclei extraction method of Galbraith et al. (1983) [67]. Hereby, fresh leaf material was chopped with a sharp razor blade in 100 μl of Galbraith’s buffer, diluted to total volume of 1.5 ml (with Galbraith’s buffer) after which the resulting nuclei suspension was filtered through a 40 μm nylon mesh. After staining the isolated nuclei with propidium iodide (final concentration of 10 μM) the corresponding DNA content was analyzed using DNA flow cytometry (excitation: 488 nm; signal detection; 575 nm).

### Availability of supporting data

The data supporting the results of this article are included within the manuscript and its additional files.

## Additional files

Additional file 1: Figure S1. Expression of *pLAT52-CENH3-GFP* in mature pollen grains. CENH3-GFP expression driven by the pollen-specific *LAT52* promoter in mature *Arabidopsis thaliana* pollen grains exhibits a strong fluorescent signal in the cytoplasm and the vegetative nucleus. Scale bar, 20 μm. (DOC 294 kb) Additional file 2: Figure S2. CENH3-GFP co-localizes with dense DAPI stained chromocenters. Expression of *p35S-CENH3-GFP* in DAPI stained guard and epidermal cells reveals that the CENH3-GFP signals co-localize with dense DAPI stained chromocenters, indicating that the CENH3-GFP fusion protein is targeted to the centromeric chromosome region. Scale bar, 5 μm. (DOC 311 kb) Additional file 3: Table S1. CENH3-GFP does not complement *cenh3* ^*−/−*^ seed lethality. Genotypic analysis of F2 progeny resulting from several F1 *cenh3-1/CENH3* plants that harbor the *pWOX2-CENH3-GFP* transgene reveals an absence of homozygous *cenh3* ^*−/−*^ plants, indicating that *cenh3* ^*−/−*^ seed fertility is not restored by the incorporation of the *pWOX2* transcribed CENH3-GFP fusion protein. (DOC 25 kb) Additional file 4: Figure S3.
*Cenh3-1/CENH3* plants harboring pWOX2-CENH3-GFP show seed abortion. Representative pictures of siliques of *Arabidopsis thaliana wild type* (A), *cenh3-1/CENH3* (B), and *cenh3-1/CENH3* plants hemizygous for the *pWOX2-CENH3-GFP* transgene (C). Heterozygous *cenh3-1/CENH3* plants harboring *pWOX2-CENH3-GFP* show a similar frequency of seed abortion as seen in the control *cenh3-1/CENH3* plants, supporting the notion that *pWOX2-CENH3-GFP* does not complement *cenh3* ^*−/−*^ seed abortion. (DOC 425 kb) Additional file 5: Figure S4. Occasional co-localization of CENH3-GFP foci during microsporogenesis. Representative images of pWOX2-CENH3-GFP fluorescence in uninuclear stage *Arabidopsis thaliana* microspores. Most microspores clearly exhibit five distinct centromeric GFP foci (A and B), reflecting the haploid chromosome number in diploid *Arabidopsis thaliana* (2x = 10). However, in some microspores, a lower number of centromeric GFP foci is observed due to the spatial co-localization or Z-axis based projection overlap of two or more GFP foci (C and D; see arrows). Scale bar, 5 μm. (DOC 151 kb) Additional file 6: Figure S5. Expression pattern of pWOX2-CENH3-GFP in early megasporogenesis. Representative images of pWOX2-CENH3-GFP fluorescence in early stage megaspores of *Arabidopsis thaliana*, displaying either the haploid number of five centromeric GFP foci in uninuclear megaspores (A, B and C) or ten GFP signals in binuclear megaspores (D; nuclei are spatially overlapping). Scale bar, 5 μm. (DOC 291 kb) Additional file 7: Figure S6. DAPI-stained chromosome spreads of 3x Arabidopsis male meiocytes. DAPI-stained chromosome spreads of somatic (A) and male meiotic (B, C and D) nuclei of triploid *Arabidopsis thaliana* plants (3x = 15). The somatic nucleus exhibits 15 distinct chromosomes, reflecting the triploid somatic chromosome number. Male meiotic chromosome spreads reveal the formation of five trivalents at diakinesis (B) and metaphase I (C), that inherently lead to an unbalanced segregation of homologous chromosomes at anaphase I (D; 8–7 chromosome segregation). Scale bar, 10 μm. (DOC 206 kb) Additional file 8: Figure S7. Frequency distribution of pWOX2-CENH3-GFP signals in nuclei of late uninuclear and binuclear megaspores isolated from triploid *Arabidopsis thaliana* plants. (DOC 54 kb) Additional file 9: Figure S8. Centromeric localization of CENH3 GFP-tailswap in different tissues. Representative images of the expression and subcellular localization of CENH3 GFP-tailswap in different tissues of *Arabidopsis thaliana*; including a young developing petal (A), a mature petal (B), unicellular stage microspores (C) and a bicellular stage microspore (E). Corresponding bright field images of the developing microspores are also presented (D and F). Scale bars, 20 μm. (DOC 241 kb) Additional file 10: Figure S9. Ploidy distribution of young and mature petals in *Arabidopsis thaliana.* Histograms representing the DNA ploidy distribution of nuclei isolated from a young, developing petal at flower stage 5–6 (A) and a mature petal at anthesis (B) of a diploid *Arabidopsis thaliana* plant. (DOC 67 kb) Additional file 11: Table S2. Sequences of primers used in this study. (DOC 25 kb)
