# Supplementary figures and images for: CENH3-GFP: a visual marker for gametophytic and somatic ploidy determination in Arabidopsis thaliana
Source: BMC Plant Biol. 2016 Jan 5;16:1. doi: 10.1186/s12870-015-0700-5 (PMC4700667; doi:10.1186/s12870-015-0700-5)

Additional file 1: Figure S1


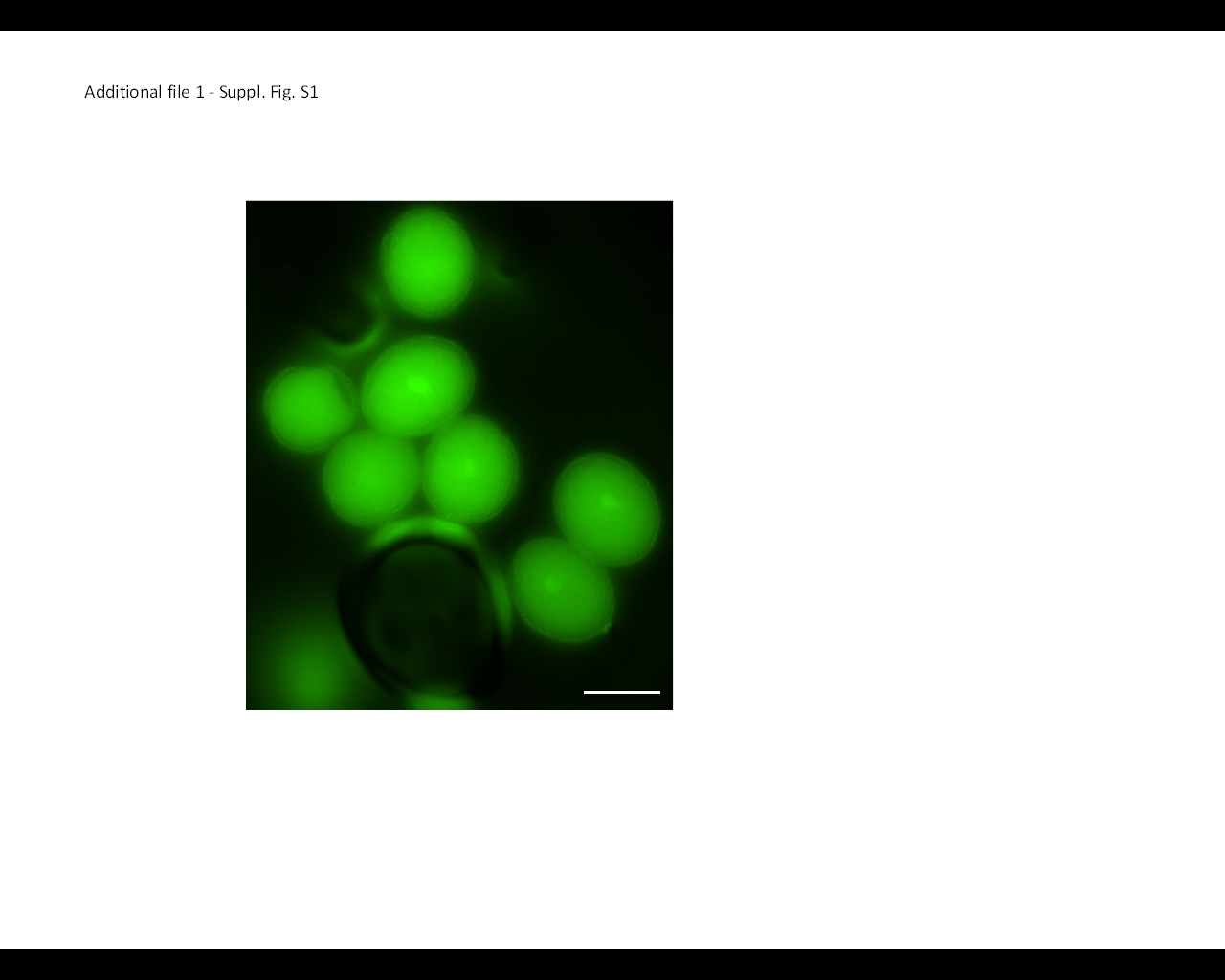

Supplement: Additional file 1: Figure S1. — Expression of pLAT52-CENH3-GFP in mature pollen grains. CENH3-GFP expression driven by the pollen-specific LAT52 promoter in mature Arabidopsis thaliana pollen grains exhibits a strong fluorescent signal in the cytoplasm and the vegetative nucleus. Scale bar, 20 μm. (DOC 294 kb) [file 12870_2015_700_MOESM1_ESM.doc]

Additional file 2: Figure S2


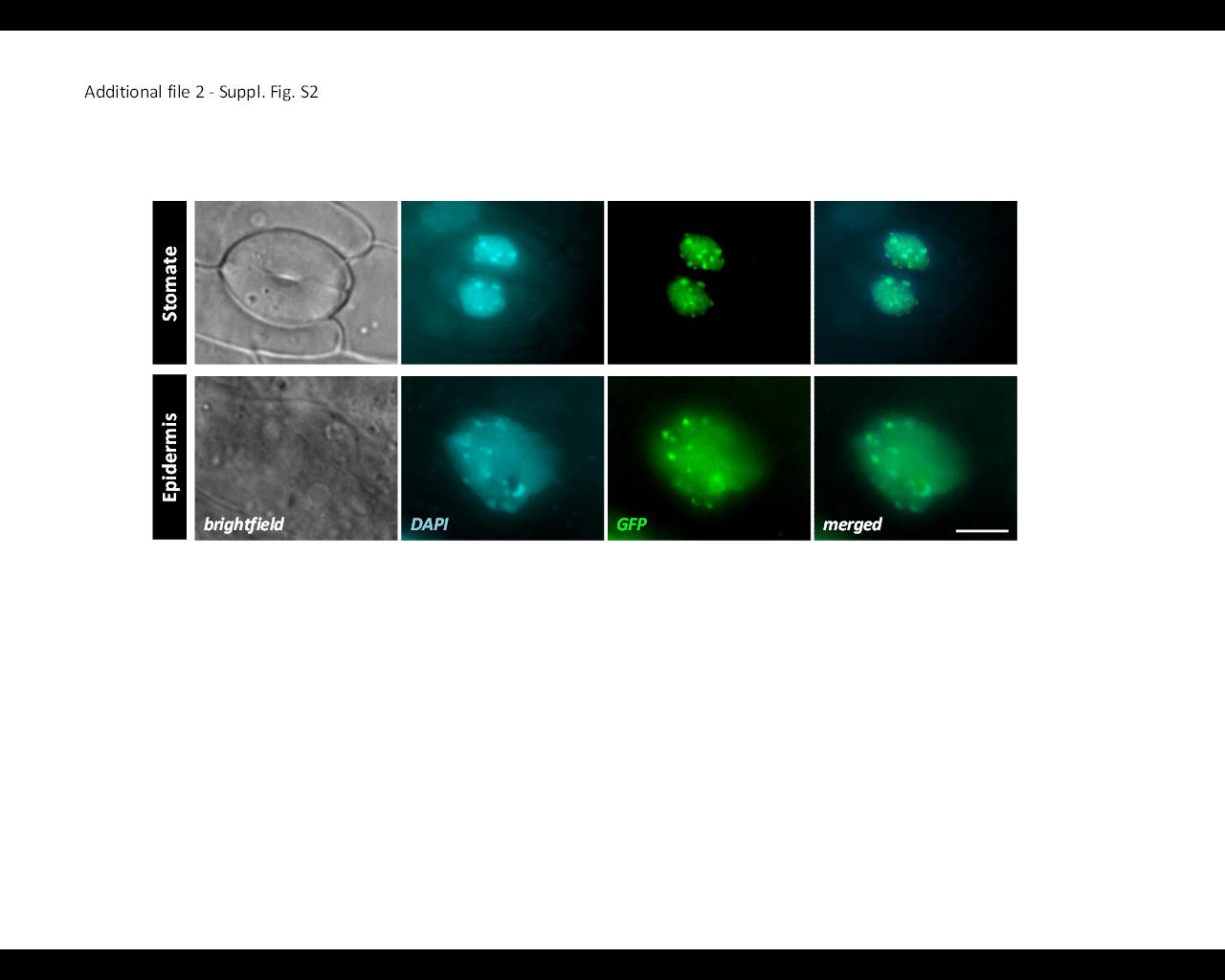

Supplement: Additional file 2: Figure S2. — CENH3-GFP co-localizes with dense DAPI stained chromocenters. Expression of p35S-CENH3-GFP in DAPI stained guard and epidermal cells reveals that the CENH3-GFP signals co-localize with dense DAPI stained chromocenters, indicating that the CENH3-GFP fusion protein is targeted to the centromeric chromosome region. Scale bar, 5 μm. (DOC 311 kb) [file 12870_2015_700_MOESM2_ESM.doc]

Additional file 5: Figure S4


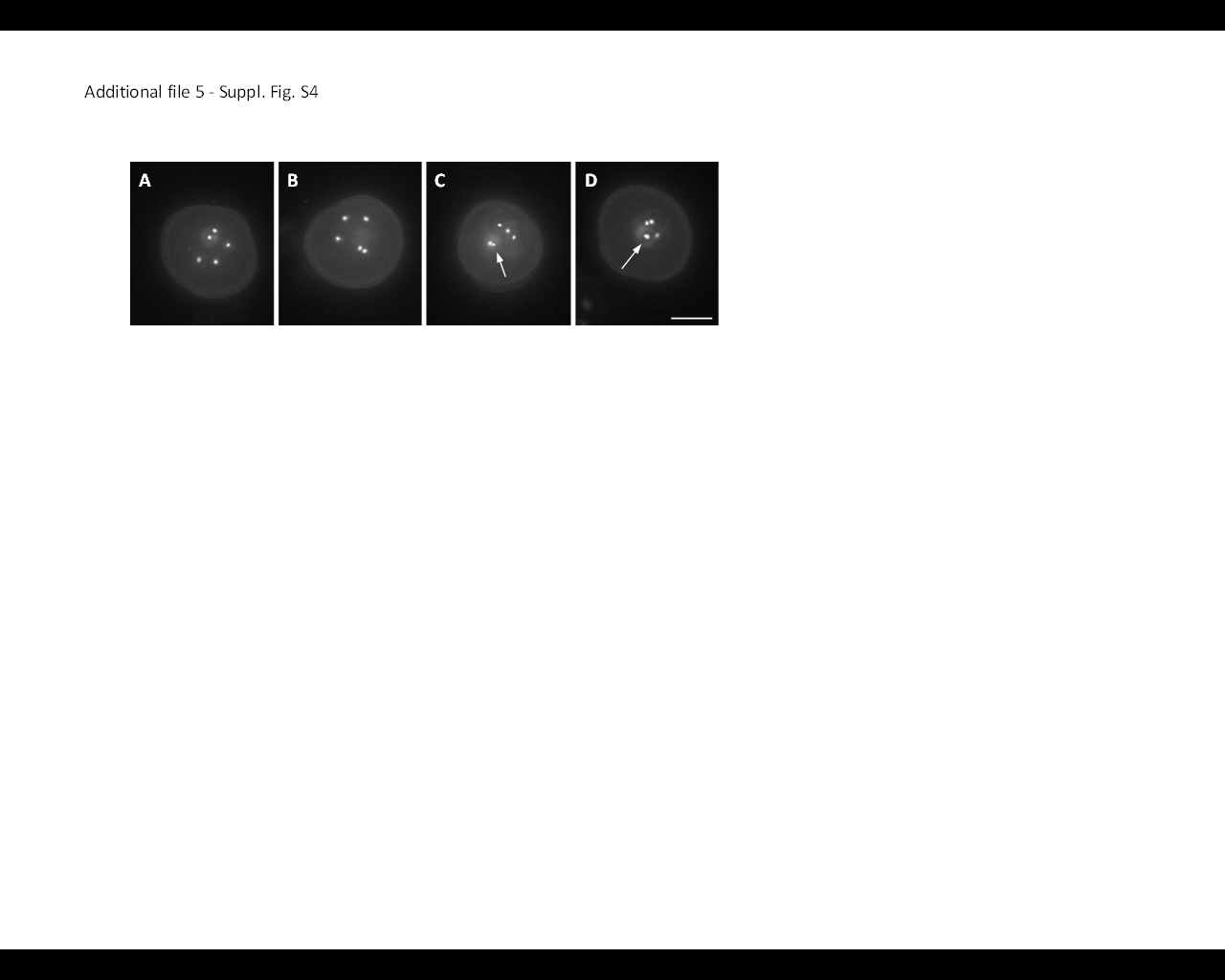

Supplement: Additional file 5: Figure S4. — Occasional co-localization of CENH3-GFP foci during microsporogenesis. Representative images of pWOX2-CENH3-GFP fluorescence in uninuclear stage Arabidopsis thaliana microspores. Most microspores clearly exhibit five distinct centromeric GFP foci (A and B), reflecting the haploid chromosome number in diploid Arabidopsis thaliana (2x = 10). However, in some microspores, a lower number of centromeric GFP foci is observed due to the spatial co-localization or Z-axis based projection overlap of two or more GFP foci (C and D; see arrows). Scale bar, 5 μm. (DOC 151 kb) [file 12870_2015_700_MOESM5_ESM.doc]

Additional file 6: Figure S5


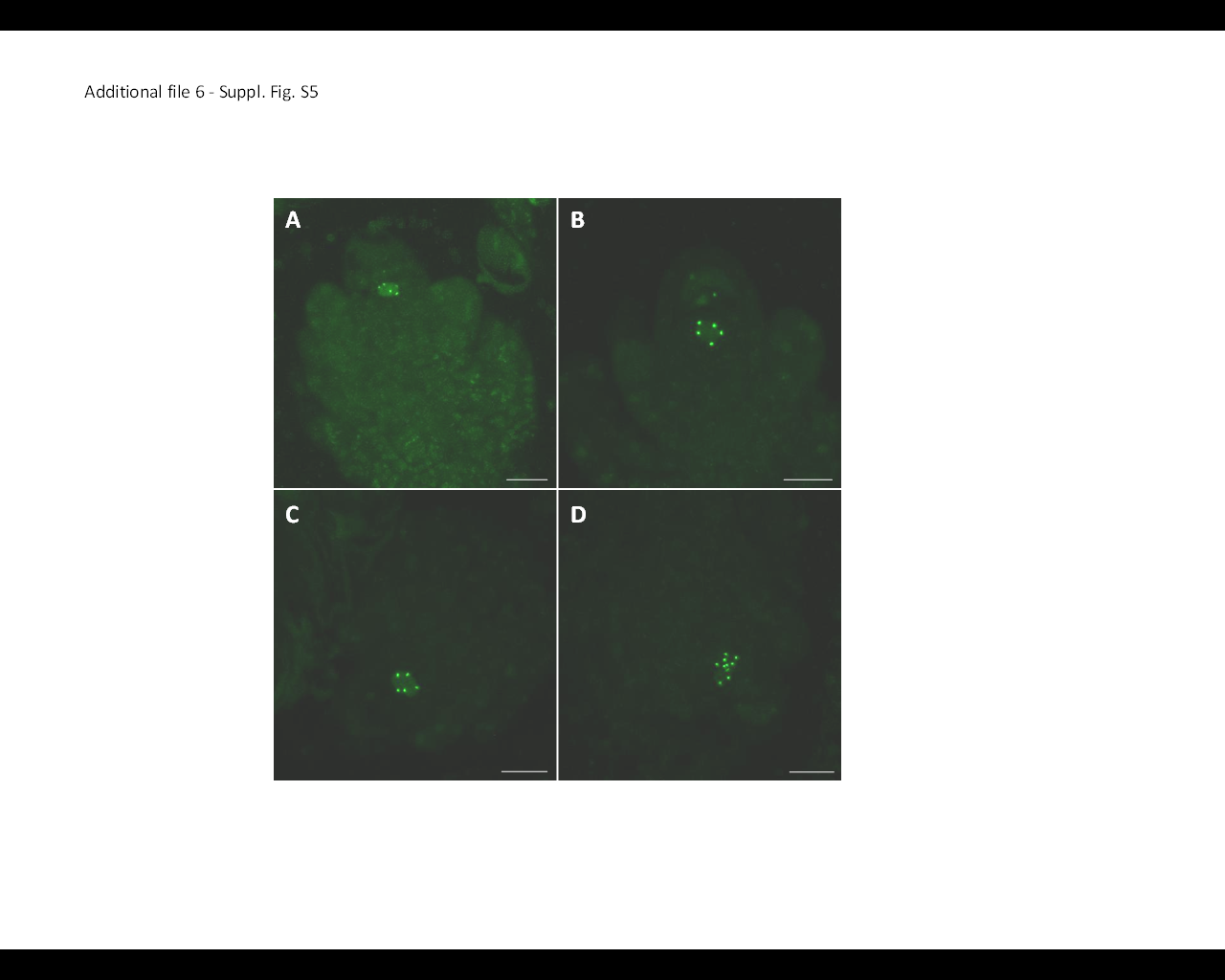

Supplement: Additional file 6: Figure S5. — Expression pattern of pWOX2-CENH3-GFP in early megasporogenesis. Representative images of pWOX2-CENH3-GFP fluorescence in early stage megaspores of Arabidopsis thaliana, displaying either the haploid number of five centromeric GFP foci in uninuclear megaspores (A, B and C) or ten GFP signals in binuclear megaspores (D; nuclei are spatially overlapping). Scale bar, 5 μm. (DOC 291 kb) [file 12870_2015_700_MOESM6_ESM.doc]

Additional file 7: Figure S6


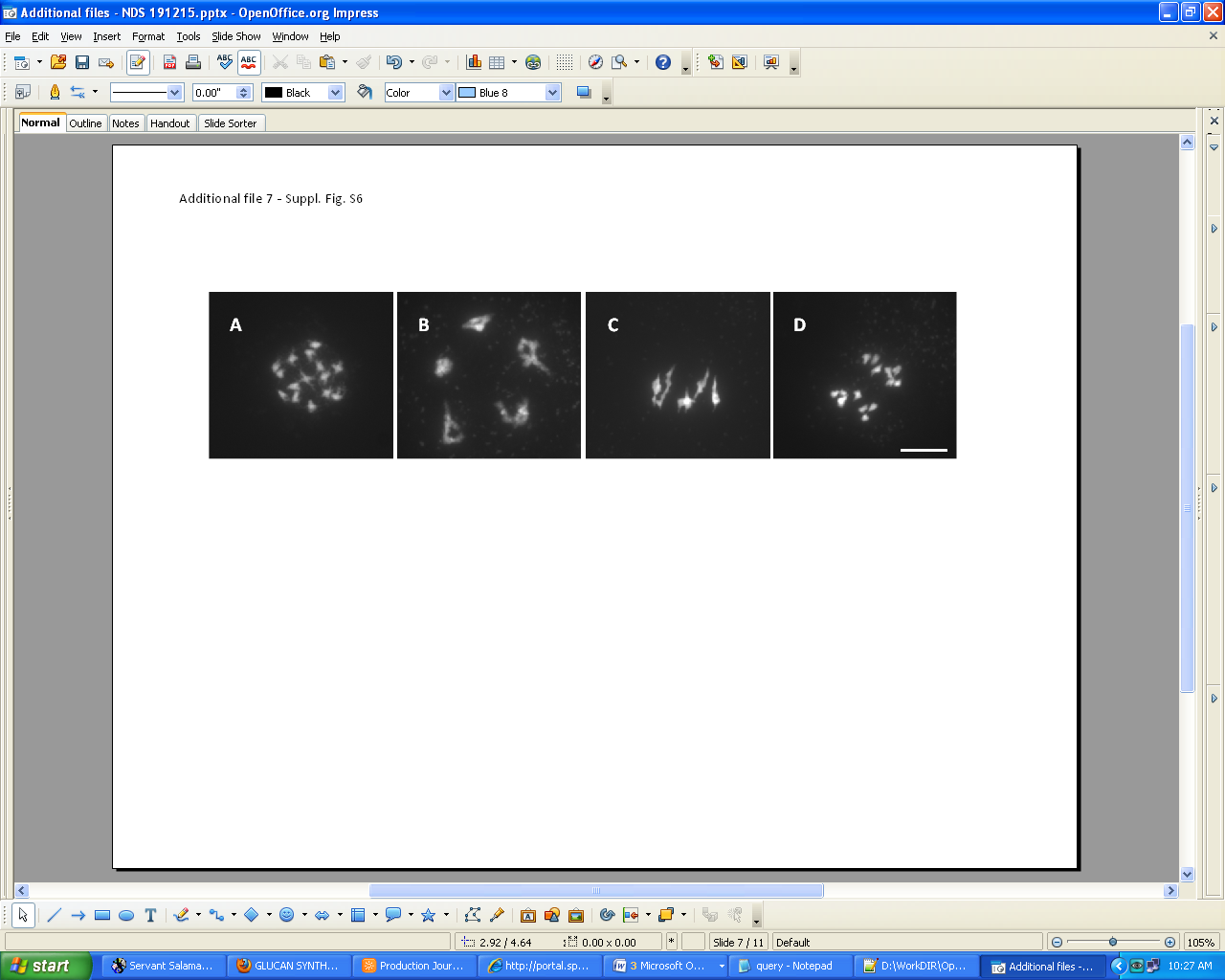

Supplement: Additional file 7: Figure S6. — DAPI-stained chromosome spreads of 3x Arabidopsis male meiocytes. DAPI-stained chromosome spreads of somatic (A) and male meiotic (B, C and D) nuclei of triploid Arabidopsis thaliana plants (3x = 15). The somatic nucleus exhibits 15 distinct chromosomes, reflecting the triploid somatic chromosome number. Male meiotic chromosome spreads reveal the formation of five trivalents at diakinesis (B) and metaphase I (C), that inherently lead to an unbalanced segregation of homologous chromosomes at anaphase I (D; 8–7 chromosome segregation). Scale bar, 10 μm. (DOC 206 kb) [file 12870_2015_700_MOESM7_ESM.doc]

Additional file 8: Figure S7


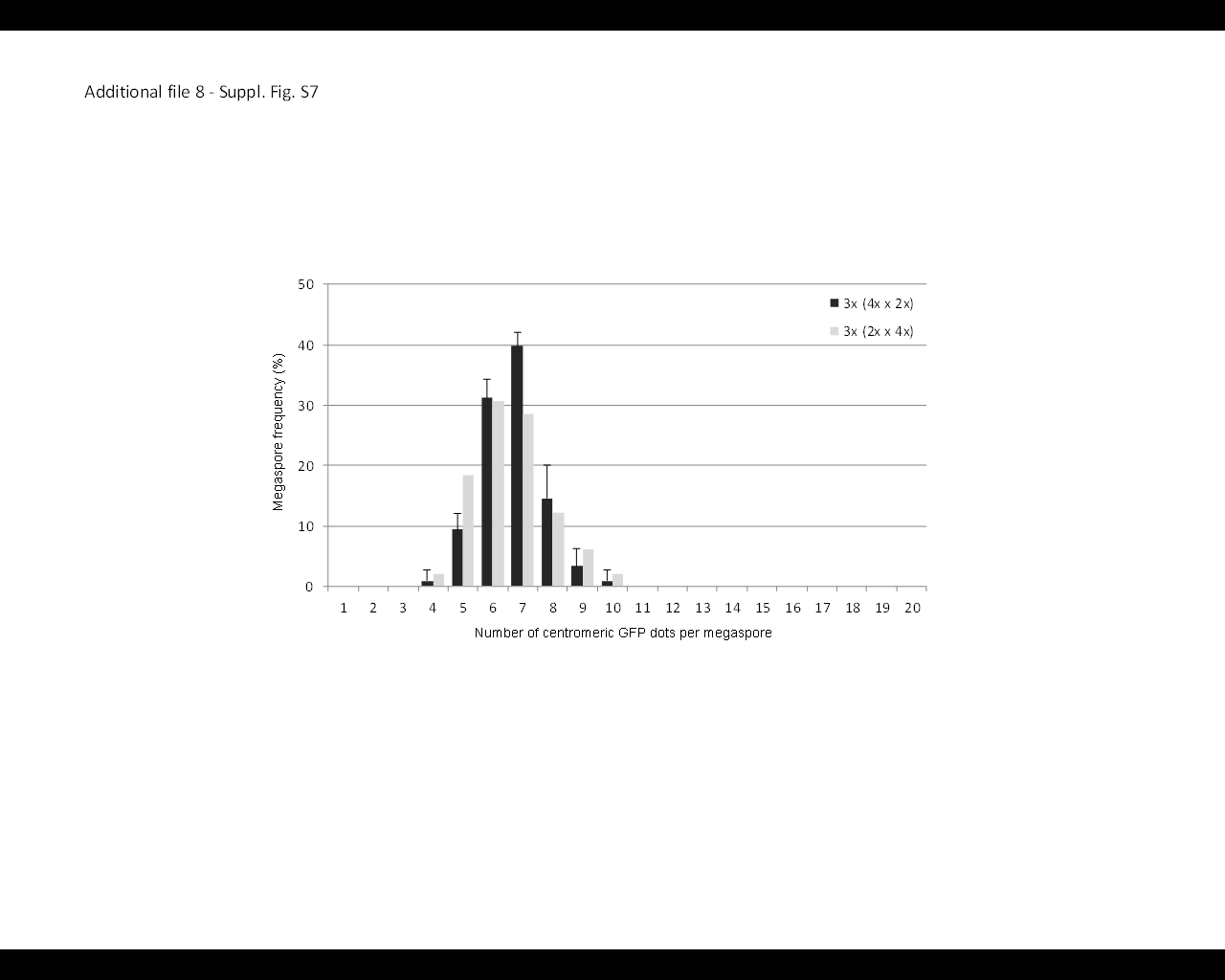

Supplement: Additional file 8: Figure S7. — Frequency distribution of pWOX2-CENH3-GFP signals in nuclei of late uninuclear and binuclear megaspores isolated from triploid Arabidopsis thaliana plants. (DOC 54 kb) [file 12870_2015_700_MOESM8_ESM.doc]

Additional file 9: Figure S8


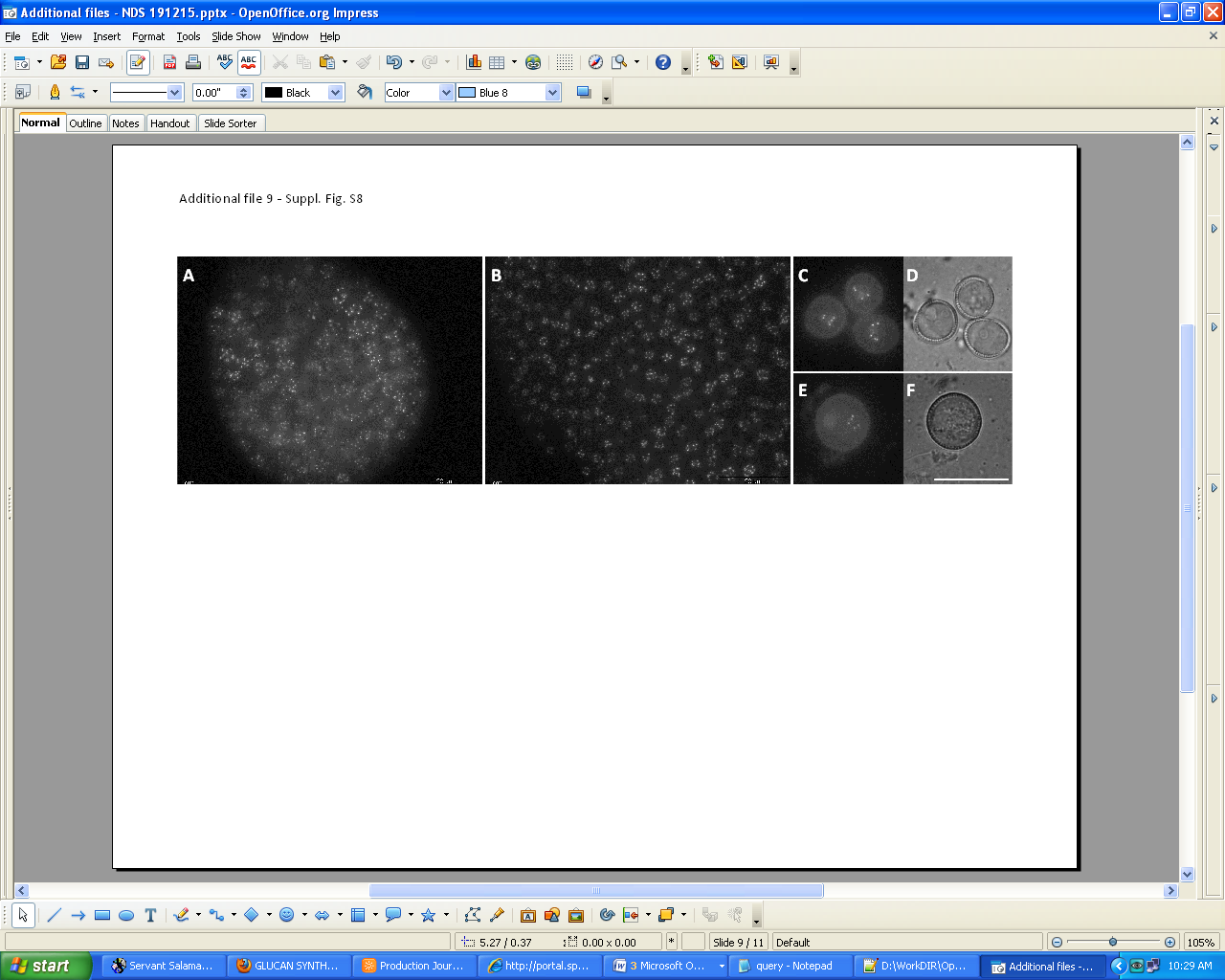

Supplement: Additional file 9: Figure S8. — Centromeric localization of CENH3 GFP-tailswap in different tissues. Representative images of the expression and subcellular localization of CENH3 GFP-tailswap in different tissues of Arabidopsis thaliana; including a young developing petal (A), a mature petal (B), unicellular stage microspores (C) and a bicellular stage microspore (E). Corresponding bright field images of the developing microspores are also presented (D and F). Scale bars, 20 μm. (DOC 241 kb) [file 12870_2015_700_MOESM9_ESM.doc]

Additional file 10: Figure S9


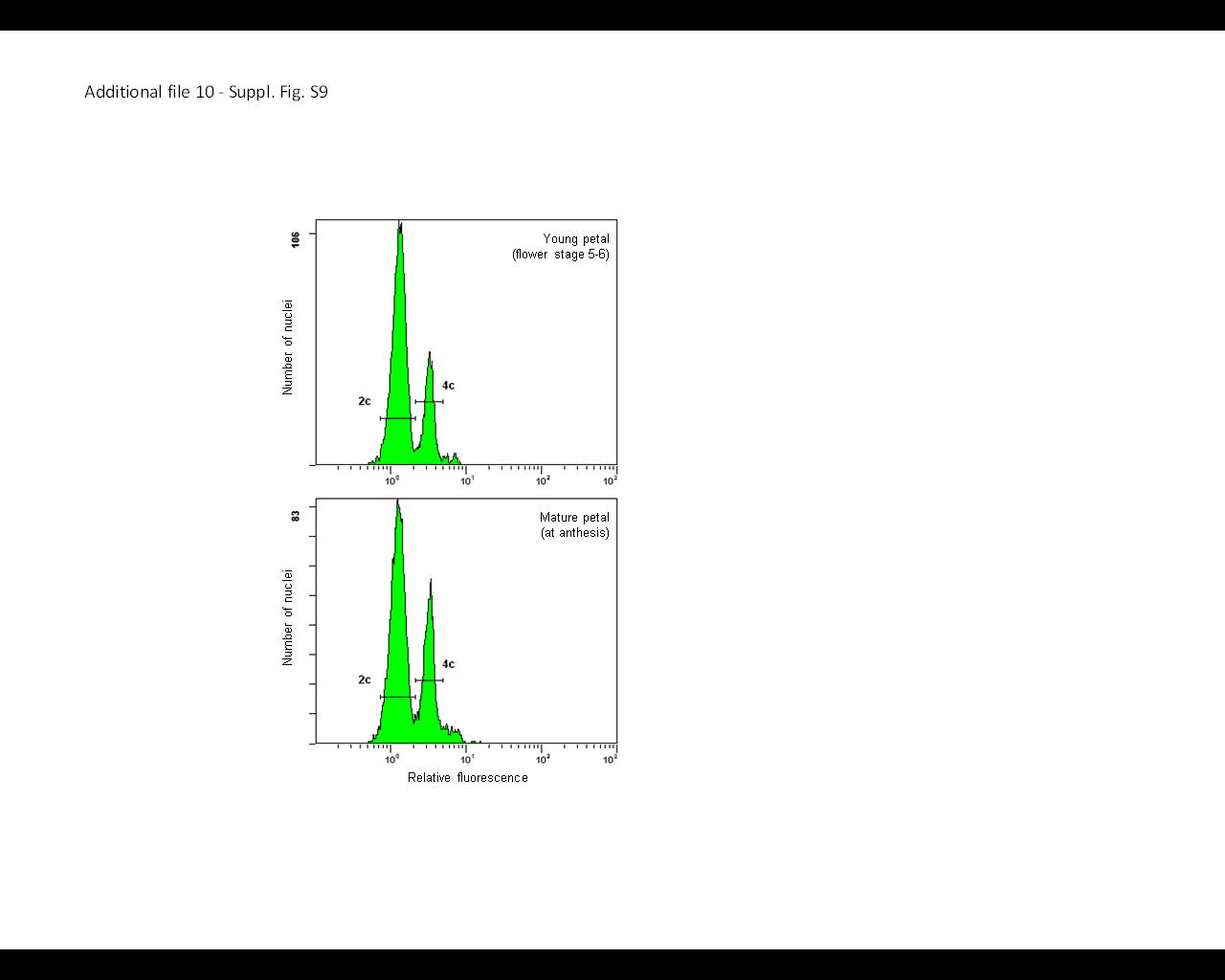

Supplement: Additional file 10: Figure S9. — Ploidy distribution of young and mature petals in Arabidopsis thaliana. Histograms representing the DNA ploidy distribution of nuclei isolated from a young, developing petal at flower stage 5–6 (A) and a mature petal at anthesis (B) of a diploid Arabidopsis thaliana plant. (DOC 67 kb) [file 12870_2015_700_MOESM10_ESM.doc]
