# Supplementary material for: CENH3-GFP: a visual marker for gametophytic and somatic ploidy determination in Arabidopsis thaliana
Source: BMC Plant Biol. 2016 Jan 5;16:1. doi: 10.1186/s12870-015-0700-5 (PMC4700667; doi:10.1186/s12870-015-0700-5)
Supplement: Additional file 3: Table S1. — CENH3-GFP does not complement cenh3 −/− seed lethality. Genotypic analysis of F2 progeny resulting from several F1 cenh3-1/CENH3 plants that harbor the pWOX2-CENH3-GFP transgene reveals an absence of homozygous cenh3 −/− plants, indicating that cenh3 −/− seed fertility is not restored by the incorporation of the pWOX2 transcribed CENH3-GFP fusion protein. (DOC 25 kb) [file 12870_2015_700_MOESM3_ESM.doc]

Additional file 3: Table S1
