# Supplementary material for: CENH3-GFP: a visual marker for gametophytic and somatic ploidy determination in Arabidopsis thaliana
Source: BMC Plant Biol. 2016 Jan 5;16:1. doi: 10.1186/s12870-015-0700-5 (PMC4700667; doi:10.1186/s12870-015-0700-5)
Supplement: Additional file 4: Figure S3. — Cenh3-1/CENH3 plants harboring pWOX2-CENH3-GFP show seed abortion. Representative pictures of siliques of Arabidopsis thaliana wild type (A), cenh3-1/CENH3 (B), and cenh3-1/CENH3 plants hemizygous for the pWOX2-CENH3-GFP transgene (C). Heterozygous cenh3-1/CENH3 plants harboring pWOX2-CENH3-GFP show a similar frequency of seed abortion as seen in the control cenh3-1/CENH3 plants, supporting the notion that pWOX2-CENH3-GFP does not complement cenh3 −/− seed abortion. (DOC 425 kb) [file 12870_2015_700_MOESM4_ESM.doc]

Additional file 4: Figure S3
