# Supplementary material for: CENH3-GFP: a visual marker for gametophytic and somatic ploidy determination in Arabidopsis thaliana
Source: BMC Plant Biol. 2016 Jan 5;16:1. doi: 10.1186/s12870-015-0700-5 (PMC4700667; doi:10.1186/s12870-015-0700-5)
Supplement: Additional file 11: Table S2. — Sequences of primers used in this study. (DOC 25 kb) [file 12870_2015_700_MOESM11_ESM.doc]

Additional file 11: Table S2
